# Engineering the oxidative myocardium: ROS-responsive biomaterials for precision cardiovascular delivery in ischemia-reperfusion injury and post-infarction remodeling

**DOI:** 10.3389/fddev.2026.1929035

**Published:** 2026-09-16

**Authors:** Hossein Omidian, Kwadwo A. Mfoafo, Luigi X. Cubeddu

**Affiliations:** 1 Department of Pharmaceutical Sciences, Barry and Judy Silverman College of Pharmacy, Nova Southeastern University, Fort Lauderdale, FL, United States; 2 School of Pharmacy, William Carey University, Biloxi, MS, United States

**Keywords:** myocardial infarction, myocardial ischemia-reperfusion injury, post-infarction remodeling, precision cardiovascular delivery, reactive oxygen species, redox-responsive biomaterials

## Abstract

Myocardial ischemia-reperfusion injury (MIRI) and post-infarction remodeling are characterized by dynamic oxidative stress that varies across myocardial regions and disease stages. Reactive oxygen species (ROS)-responsive and redox-modulating biomaterials seek to exploit this environment through stimulus-triggered release or degradation, ROS scavenging, targeted delivery, local retention, and stage-specific repair. This review critically evaluates preclinical strategies for MIRI, myocardial infarction, and post-infarction remodeling. Major platform classes differ substantially in mechanism and translational feasibility: injectable hydrogels offer local retention and spatiotemporal control but often require invasive administration; systemic nanoparticles and liposomes enable tissue-, cell-, or organelle-level targeting but require stronger biodistribution and clearance data; nanozymes provide sustained catalytic redox activity but raise persistence and safety concerns; and cell-, nucleic-acid-, or gas-based systems introduce additional manufacturing and regulatory complexity. Mechanistically, redox interaction represents the initiating action, followed by proximal mitochondrial and cell-death regulation, inflammatory modulation, and later effects on angiogenesis, fibrosis, and ventricular remodeling. Although preclinical studies demonstrate cardioprotective and repair-promoting effects, direct cross-platform comparisons remain limited. Translation is constrained by heterogeneous models, inconsistent definitions of ROS responsiveness, incomplete pharmacokinetic and safety characterization, sparse large-animal validation, and limited manufacturing evidence. ROS-responsive cardiovascular biomaterials therefore remain promising but clinically unproven precision-delivery strategies.

## Graphical Abstract

**Figure:**
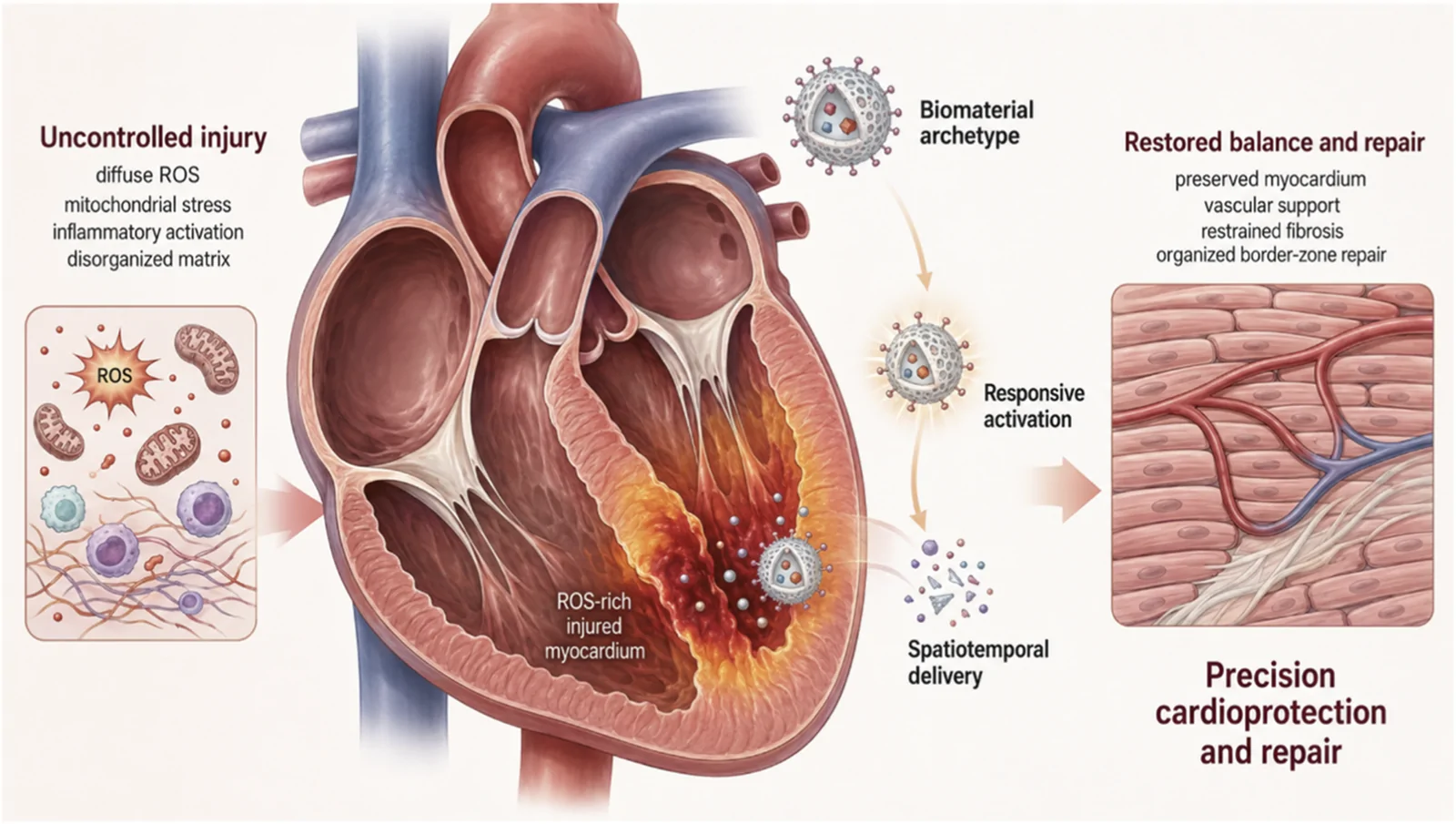
Prepared with the assistance of Gemini NotebookLM pro and OpenAI ChatGPT Plus.

## Introduction

Myocardial ischemia-reperfusion injury and post-infarction remodeling are pathobiologically related but therapeutically distinct stages of ischemic heart disease. Reperfusion is essential for myocardial salvage, yet restoration of blood flow can intensify oxidative stress, mitochondrial dysfunction, inflammatory signaling, and regulated cardiomyocyte death (Hu et al., 2026; Qin et al., 2026; Wang et al., 2025c; Wang et al., 2026). The resulting redox disturbance is neither spatially uniform nor temporally fixed. It evolves from the acute reperfusion phase through inflammatory resolution, vascular repair, scar formation, and ventricular remodeling, while also differing between injury core and border regions (Song L. et al., 2025; Zhen et al., 2025). This heterogeneity creates a rationale for delivery systems that use pathological redox conditions not only as a therapeutic target but, in selected designs, as a trigger that controls when and where a material releases its cargo.

The term ROS-responsive, however, covers mechanistically different technologies. Some systems use ROS as an active trigger for bond cleavage, material degradation, or payload release (Cao et al., 2026; Hu et al., 2026; Qin et al., 2026; Song L. et al., 2025; Wang Y. et al., 2024; Zhen et al., 2025). Others primarily scavenge ROS, catalytically convert reactive species, deliver antioxidants to mitochondria or inflammatory cells, generate oxygen, release redox-active gases, or modulate endogenous antioxidant and immunometabolic pathways (Ai et al., 2021; Cheng et al., 2019; Fu et al., 2026; Hao et al., 2022; Hu et al., 2024; Wang et al., 2025c; Wang et al., 2026; Xu et al., 2026; Xue et al., 2026; Zhu et al., 2025). Therapeutic reduction of ROS should therefore not be treated as proof of ROS-responsive material behavior. A rigorous synthesis must distinguish true stimulus-responsive delivery from direct antioxidant or redox-regulatory therapy.

The major material classes also involve different delivery trade-offs. Local hydrogels and microgels can improve myocardial residence and support staged or gradient-sensitive release (Song L. et al., 2025; Wang et al., 2022; Zhen et al., 2025), but direct myocardial administration can limit procedural applicability. Systemically delivered nanoparticles and liposomes may be more compatible with narrow peri-reperfusion treatment windows and can incorporate tissue-, cell-, or organelle-level targeting (Ikeda et al., 2016; Li et al., 2026; Ma X. B. et al., 2025; Wang et al., 2025c; Wang Y. et al., 2024), but their translational value depends on quantitative biodistribution, off-target exposure, and clearance. Catalytic nanozymes may provide sustained or repeated ROS detoxification (Fu et al., 2026; Gong et al., 2025; Wang et al., 2025c; Xu et al., 2026; Zhu et al., 2025), while simultaneously raising questions about inorganic persistence, particle transformation, and long-term material safety. Cell-, nucleic-acid-, membrane-derived, and gas-delivery systems expand therapeutic functionality but introduce additional manufacturing, immunologic, pharmacologic, or regulatory complexity (Cao et al., 2026; Chen X. R. et al., 2025; Hao et al., 2022; Liao et al., 2025; Wang et al., 2025b; Wang et al., 2026; Wang Y. et al., 2025; Wu C. R. et al., 2025; Hu et al., 2026).

Mechanistic interpretation likewise requires hierarchy. The primary event may be ROS-triggered release, ROS consumption, catalytic conversion, mitochondrial drug delivery, or direct redox-pathway modulation. Proximal cellular responses include stabilization of mitochondrial membrane potential, inhibition of mitochondrial permeability transition pore (mPTP) opening, calcium regulation, and suppression of apoptosis, ferroptosis, pyroptosis, or cuproptosis (Cheng et al., 2019; Hu et al., 2026; Ikeda et al., 2016; Wang et al., 2025c; Wang et al., 2026; Wang Y. et al., 2024). Inflammatory changes may occur downstream of reduced tissue injury or may be targeted directly through macrophage-, monocyte-, or immune-directed systems (Hu et al., 2024; Liao et al., 2025; Pan et al., 2025; Xue et al., 2026). Angiogenesis, fibrosis, scar formation, ventricular geometry, and cardiac function are later tissue-level outcomes. This distinction is not merely semantic: mitochondria-targeted cyclosporine A nanoparticles reduced infarction and improved 28-day remodeling without altering inflammatory monocyte recruitment, demonstrating that mitochondrial protection can improve cardiac outcome without obligatory suppression of this inflammatory compartment (Ikeda et al., 2016).

The evidence base remains predominantly preclinical. Most cardiac studies use rodent models, while only one explicitly identified study extended evaluation to a porcine MI model (Wang et al., 2025b). No human observational or clinical-trial evidence was identified in the included evidence base. Accordingly, this review evaluates ROS-interacting cardiovascular biomaterials through five questions: whether ROS responsiveness is quantitatively demonstrated; whether a carrier improves delivery relative to an appropriate comparator; which mechanisms are primary versus downstream; whether benefit persists into functional or remodeling outcomes; and whether safety, clearance, administration, and manufacturability support a plausible translational pathway.

### Evidence identity, scope, and eligibility

#### Evidence scope and hierarchical classification

The evidence was organized according to disease relevance and evidentiary proximity rather than treating all redox-related studies as equivalent. Primary cardiac evidence comprised studies directly involving MIRI or reperfusion-associated myocardial injury, acute myocardial ischemia, MI, ST-elevation myocardial infarction (STEMI), infarct repair, fibrosis, angiogenesis, or post-infarction ventricular remodeling. Cardiac studies using non-coronary oxidative injury models were retained as secondary mechanistic evidence (Bei et al., 2020; Boarescu et al., 2019; Liu Z. C. et al., 2026; Zhao et al., 2026). Studies confined to non-cardiac organs were used only for cross-organ material or mechanistic context. This hierarchy is important because efficacy in another organ does not establish myocardial targeting, cardiovascular pharmacokinetics, ventricular benefit, electrophysiological safety, or cardiac material tolerability.

#### Primary cardiac evidence

The primary cardiac synthesis includes 74 preclinical studies. Operationally, 30 addressed MIRI or reperfusion-associated myocardial injury, represented by studies of antioxidant nanoparticles, ROS-responsive hydrogels, theranostic nanoplatforms, mitochondrial delivery systems, and hierarchical nanozymes (Altshuler et al., 2021; Hu et al., 2026; Ma X. B. et al., 2025; Wang et al., 2025c; Wang Y. et al., 2024; Zhen et al., 2025; Zhu et al., 2025). The remaining 44 addressed MI, acute ischemia, infarct repair, fibrosis, angiogenesis, or post-MI remodeling, represented by local hydrogel, patch, immunomodulatory, regenerative, and antifibrotic strategies (Cao et al., 2026; Song L. et al., 2025; Taiwaikuli et al., 2026; Wang et al., 2025b; Wang et al., 2022; Yang J. Q. et al., 2025; Zhu et al., 2024). These categories identify the dominant experimental context and are not mutually exclusive biological stages; several reperfusion studies included longer-term remodeling outcomes, and several MI studies examined acute injury responses.

Two studies required component-level classification because they included both cardiac and non-cardiac ischemic models. One study contributed direct cardiac I/R evidence together with a hepatic I/R arm (Lee et al., 2015), whereas another study included both MI and stroke models (Wang et al., 2024d). Their cardiac components were retained in the primary synthesis and their non-cardiac components were treated only as cross-organ evidence.

#### Evidence-level classification and translational weight

Within the evidence extraction used for this review, 57 of the 74 primary cardiac studies were explicitly coded as mouse, rat, or murine *in vivo* models; species or experimental scale was not available in the remaining extracted records. Cellular systems were frequently used alongside *in vivo* experiments (Dai et al., 2026; Hu et al., 2024; Li et al., 2026; Qin et al., 2026; Wang et al., 2025c; Zhang et al., 2025; Altshuler et al., 2021). One study additionally included *ex vivo* regional I/R experiments in isolated rat hearts, providing an intermediate mechanistic model but not evidence of systemic pharmacokinetics or chronic safety (Liu et al., 2016). The only explicitly identified large-animal cardiac study combined mouse experiments with a porcine MI model (Wang et al., 2025b). A single large-animal study strengthens the evidence for that platform but does not establish reproducibility, dose scaling, chronic safety, or clinical readiness across the field.

#### Secondary cardiac and cross-organ evidence

Four cardiac studies were retained as secondary evidence because their injury models were not direct coronary ischemia-reperfusion models: isoproterenol-associated myocardial injury in (Bei et al., 2020; Boarescu et al., 2019; Zhao et al., 2026) and a sepsis/LPS-associated myocardial injury model in (Liu Z. C. et al., 2026). These studies can support antioxidant, material, electrophysiological, or myocardial-repair mechanisms but should not substitute for direct MIRI or MI evidence.

Twenty-two studies were exclusively non-cardiac (Afshar et al., 2024; Deng et al., 2026; Embaby et al., 2026; Feng et al., 2022; Guo et al., 2021; Hu et al., 2025; Jia et al., 2025; Lee et al., 2026; Li et al., 2016; Li et al., 2025; Li et al., 2022; Liu et al., 2022; Liu et al., 2020; Lu et al., 2020; Ma J. et al., 2025; Park et al., 2026; Sheng et al., 2025; Shin et al., 2022; You et al., 2024; Zhang et al., 2026; Zheng et al., 2025; Zhou et al., 2025). Together with the non-cardiac arms of (Lee et al., 2015; Wang Z. C. et al., 2024), these studies can inform general principles such as ROS-triggered degradation, catalytic scavenging, uptake, imaging, or material safety. They cannot be used to infer myocardial accumulation, cardiac pharmacokinetics, ventricular efficacy, arrhythmia safety, or post-infarction remodeling.

#### Evidence boundary and overall interpretation

The 100-study evidence base therefore comprises 74 primary cardiac studies, four secondary cardiac studies, and 22 exclusively non-cardiac studies, with mixed-organ components handled at the level of the relevant model. No human evidence was identified. Stronger conclusions in this review preferentially rely on direct cardiac models, appropriate carrier or free-drug comparators, quantitative delivery or responsiveness measurements, and functional or remodeling endpoints; secondary and cross-organ studies are used only for mechanism, material, or safety context. The evidence categories and their roles in the synthesis are summarized in Table 1, while Figure 1 depicts the corresponding evidence hierarchy and translational depth.

**TABLE 1:** Evidence identity, scope, and eligibility of the cardiovascular synthesis.

Evidence category	Number	Role in synthesis	Representative references
Primary MIRI/reperfusion-associated myocardial injury	30	Direct evidence for reperfusion-stage redox-responsive or redox-modulating cardiovascular therapy	(Ai et al., 2021; Altshuler et al., 2021; Cheng et al., 2019; Hu et al., 2026; Ikeda et al., 2016; Liu et al., 2016; Ma et al., 2025b; Wang et al., 2025c; Wang et al., 2024c; Zhen et al., 2025)
Primary MI/post-infarction evidence	44	Direct evidence for acute MI, infarct repair, angiogenesis, fibrosis, and remodeling	(Cao et al., 2026; Ding et al., 2022; Ding et al., 2020; Ji et al., 2023; Pan et al., 2025; Song L. et al., 2025; Wang et al., 2025b; Wang et al., 2022; Wu et al., 2025a; Yang et al., 2025a)
Secondary cardiac evidence	4	Mechanistic cardiac evidence from non-coronary oxidative injury models	(Bei et al., 2020; Boarescu et al., 2019; Liu et al., 2026b; Zhao et al., 2026)
Exclusively non-cardiac evidence	22	Cross-organ material, mechanistic, imaging, or safety context only	(Afshar et al., 2024; Deng et al., 2026; Embaby et al., 2026; Feng et al., 2022; Hu et al., 2025; Ma et al., 2025a; Sheng et al., 2025; You et al., 2024; Zhang et al., 2026; Zhou et al., 2025)
Human evidence	0	No human observational or clinical-trial evidence identified	None

**FIGURE 1:**
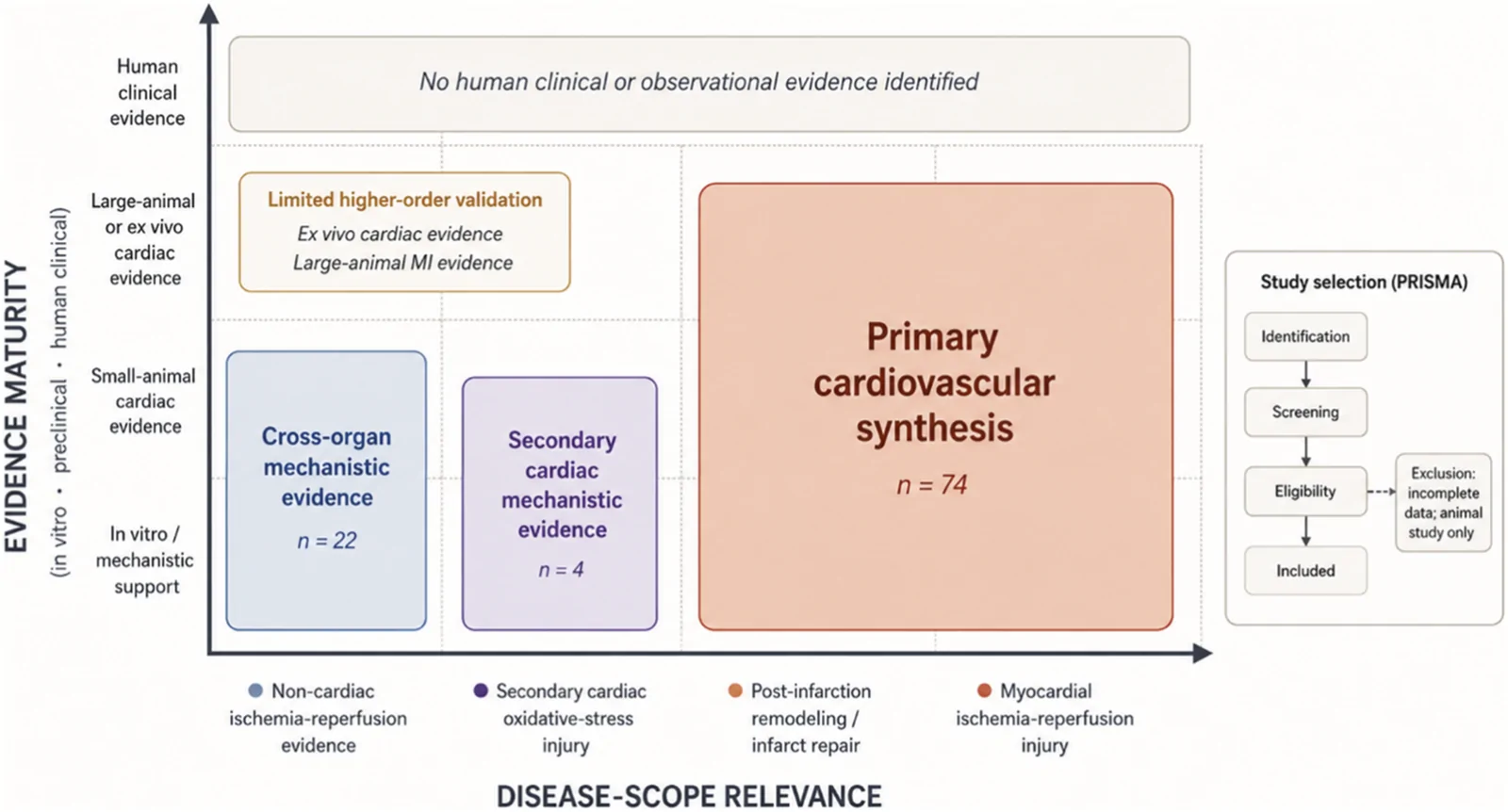
Evidence hierarchy and translational depth of the included literature. The primary synthesis comprises 74 cardiac preclinical studies, supplemented by four secondary cardiac studies and 22 exclusively non-cardiac studies (*Prepared with the assistance of Gemini NotebookLM pro and OpenAI ChatGPT Plus*).

### Disease context and experimental model

#### Model identity determines the meaning of platform performance

The experimental literature spans pretreatment or prevention protocols, acute myocardial ischemia, early reperfusion injury, established MI, subacute repair, fibrosis, and chronic ventricular remodeling (Ai et al., 2021; Ikeda et al., 2016; Shi et al., 2024; Wang et al., 2025b; Wang et al., 2022; Weng et al., 2022; Zhen et al., 2025). These settings are not interchangeable. A platform that performs well during a brief reperfusion-associated oxidative burst is not automatically optimized for a permanent infarct, and a long-lived local depot designed for fibrosis control may be poorly suited to the narrow treatment window of acute reperfusion. Cross-study effect sizes should therefore be interpreted in the context of model identity, injury severity, treatment timing, route, and follow-up rather than as direct measures of relative material superiority.

#### Pre-injury and pre-ischemic prevention models

Several studies used preventive or pre-ischemic dosing. Examples include oral nanoparticle regimens before isoproterenol-associated myocardial injury (Bei et al., 2020; Boarescu et al., 2019) and lutein@DTPP administered once daily for 3 days before 45 min of ischemia followed by 6 h of reperfusion (Shi et al., 2024). Such models are valuable for demonstrating mechanism and prophylactic capacity, but they differ clinically from treatment initiated at reperfusion or after MI is established. Preventive efficacy should therefore not be generalized to an emergency-treatment setting without post-injury validation.

#### Acute ischemia and early reperfusion injury

Acute MIRI models provide the most direct setting for testing rapid redox control and peri-reperfusion delivery. Bilirubin nanoparticles were evaluated after 45 min of LAD occlusion with dosing 5 min before and again 24 h after reperfusion (Ai et al., 2021). TPE-ss COF@Matrine was administered 5 min after a 30-min ischemic interval (Huang et al., 2022), whereas mitochondria-targeted CsA nanoparticles were administered intravenously at the onset of reperfusion and remodeling was followed for 28 days (Ikeda et al., 2016). These differences in ischemia duration, treatment timing, and follow-up illustrate why apparently similar MIRI studies can test different therapeutic questions.

Context can also alter apparent treatment effect. In the circadian HA-RES-OPC-MMP study, injury was less severe at ZT13 than at ZT1 and nanoparticle efficacy was correspondingly smaller; Per1/2 knockout experiments supported rhythm dependency (Zhang et al., 2024). A smaller observed therapeutic effect therefore need not indicate an inferior material if the untreated injury context is itself milder.

#### Myocardial infarction and subacute repair

Established MI models shift the therapeutic emphasis from immediate reperfusion injury toward infarct stabilization, inflammatory resolution, angiogenesis, and prevention of adverse remodeling. Permanent LAD ligation was used in the CeO2/Nrf2 nanocomposite study (Liao et al., 2025). PVAX-NPY3-36 was evaluated after LAD ligation in 49 randomized C57BL/J6 mice over 2 weeks (Mahmood et al., 2020), while LDE-MTX was compared with empty LDE and commercial methotrexate after left coronary ligation in Wistar rats with echocardiographic follow-up to 6 weeks (Maranhao et al., 2017). These designs support later repair-oriented questions that differ from acute transient-occlusion models.

#### Post-infarction fibrosis and chronic remodeling

Longer follow-up is essential when the therapeutic claim concerns fibrosis or ventricular remodeling. R gel was compared with a non-reactive hydrogel at 3 days and 8 weeks (Wang et al., 2022). SaB/Cand microgels linked short-term anti-inflammatory activity with longer-term antifibrotic effects and reduced ventricular dilation (Song L. et al., 2025). The miR-19a/b ROS-eliminating hydrogel was evaluated in a porcine MI model over 50 days (Wang et al., 2025b). These studies provide more relevant evidence for durable post-infarction repair than short-term ROS or apoptosis measurements alone.

#### Cellular and ex vivo experimental systems

*In vitro* and *ex vivo* systems provide mechanistic resolution but model different biological stresses. Hypoxia/reoxygenation assays, direct H2O2 exposure, oxygen-glucose deprivation, and macrophage activation experiments should not be treated as equivalent representations of myocardial I/R. Convergence across these systems can strengthen mechanistic plausibility, whereas disagreement may reflect the different stressor rather than a true contradiction. Study (Liu et al., 2016) adds isolated-heart regional I/R evidence to its *in vivo* rat experiments, but *ex vivo* cardiac preparations cannot reproduce systemic biodistribution, circulating immunity, clearance, or chronic material effects.

#### Comparator quality and reporting context

The strongest experimental designs isolate the contribution of a specific delivery feature. Examples include CsA nanoparticles versus free CsA (Ikeda et al., 2016), fibroblast-targeted PF543 nanoparticles versus systemic drug administration (Ji et al., 2023), R gel versus a non-reactive hydrogel (Wang et al., 2022), LDE-MTX versus empty LDE and commercial methotrexate (Maranhao et al., 2017), MitoQ hydrogel delivered by intramyocardial injection versus myocardial surface patch (Tan et al., 2024), and NO-RIG versus simpler NO-releasing or ROS-scavenging hydrogels (Vong et al., 2018). These comparisons are more informative than treatment-versus-saline designs when the aim is to attribute benefit specifically to targeting, responsiveness, route, or multifunctionality.

Reporting characteristics were heterogeneous in the evidence extraction. Explicit randomization was captured for study (Mahmood et al., 2020), whereas blinding information was not captured. Sex was explicitly stated in selected cardiac studies, including male animals in (Bei et al., 2020; Sun et al., 2023). Because absence from the extraction does not establish omission from the original publication, these observations are treated as limitations of the available reporting data rather than definitive study-level deficiencies. Future comparative studies should report randomization, allocation, blinding, sex, age, comorbidities, and prespecified analysis consistently.

The relationship among disease stage, experimental model, intervention window, and evidentiary depth is summarized in Table 2 and Figure 2.

**TABLE 2:** Experimental contexts and their implications for interpreting ROS-interacting cardiovascular biomaterials.

Experimental context	Primary question	Strength	Main limitation	Representative refs
Pretreatment/prevention	Can the platform prevent oxidative injury before or around onset?	Mechanistically useful for prophylactic protection	Limited applicability to unplanned acute MI or reperfusion therapy	(Bei et al., 2020; Boarescu et al., 2019; Shi et al., 2024)
Acute transient ischemia-reperfusion	Can rapid redox intervention limit reperfusion injury?	Directly relevant to MIRI biology	Sensitive to ischemia duration and dosing window	(Ai et al., 2021; Huang et al., 2022; Ikeda et al., 2016; Ma et al., 2025b; Wang et al., 2025c; Wang et al., 2024c; Zhen et al., 2025)
Established MI/permanent ligation	Can treatment improve infarct repair after injury is established?	Relevant to repair, angiogenesis, and remodeling	Not equivalent to reperfused MI	(Liao et al., 2025; Mahmood et al., 2020; Maranhao et al., 2017; Pan et al., 2025)
Chronic remodeling follow-up	Are structural and functional benefits durable?	Captures fibrosis, dilation, and long-term function	Often lacks parallel chronic material-safety data	(Song L. et al., 2025; Wang et al., 2025b; Wang et al., 2022; Wang et al., 2024b; Yang et al., 2025a; Zheng et al., 2024)
Cellular stress assays	Which molecular pathways are affected?	Mechanistic resolution	H/R, H_2_O_2_, OGD, and immune assays are not equivalent	(Altshuler et al., 2021; Dai et al., 2026; Hu et al., 2024; Li et al., 2026; Qin et al., 2026; Wang et al., 2025c; Zhang et al., 2025)
*Ex vivo* heart	Does cardioprotection occur in intact cardiac tissue without systemic confounding?	Intermediate between cells and *in vivo* models	No systemic PK, immunity, or clearance	(Liu et al., 2016)
Direct route comparison	How much does administration route alter efficacy?	Controls material and payload while changing route	Rarely performed	(Tan et al., 2024)
Mixed-organ ischemic models	Are redox/material principles transferable across tissues?	Useful mechanistic context	Cannot establish cardiac delivery or safety	(Lee et al., 2015; Wang et al., 2024d)

**FIGURE 2:**
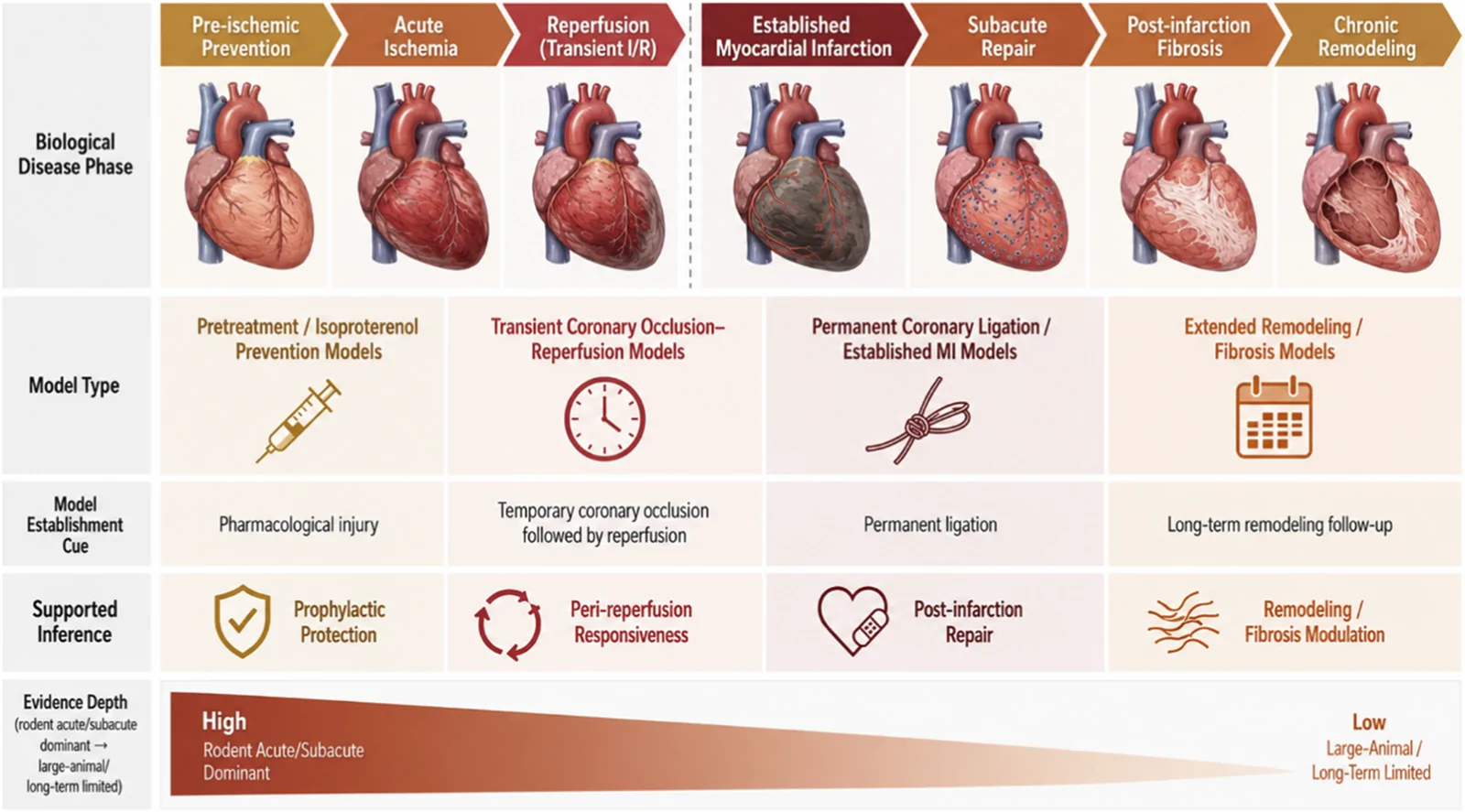
Experimental disease contexts used to evaluate ROS-responsive cardiovascular biomaterials. The figure aligns biological disease chronology with pretreatment, acute transient ischemia-reperfusion, established MI, and chronic remodeling models. The separation between reperfused and non-reperfused injury emphasizes that these settings test different delivery and therapeutic questions. Evidence-depth indicators distinguish predominantly rodent studies from the limited *ex vivo* and large-animal evidence (*Prepared with the assistance of Gemini NotebookLM pro and OpenAI ChatGPT Plus*).

### Intervention design and ROS-Interaction mechanism

#### A functional rather than material-based classification

The interventions reviewed here should not be grouped solely by whether the carrier is a hydrogel, nanoparticle, liposome, or nanozyme. A more mechanistically informative classification asks how ROS or redox biology is used. Five principal strategies are evident. First, ROS-triggered or redox-labile systems use oxidative conditions to initiate bond cleavage, matrix degradation, or payload release (Cao et al., 2026; Hu et al., 2026; Qin et al., 2026; Song L. et al., 2025; Wang Y. et al., 2024; Zhen et al., 2025). Second, direct ROS-scavenging systems neutralize oxidative species or improve delivery of antioxidant therapeutics without necessarily demonstrating stimulus-dependent release (Ai et al., 2021; Cheng et al., 2019; Hu et al., 2024; Liao et al., 2022; Tan et al., 2024). Third, catalytic nanozymes or enzyme-delivery systems seek sustained ROS conversion through SOD-, catalase-, peroxidase-, or related enzyme-mimetic activity (Altshuler et al., 2021; Chen X. R. et al., 2025; Dai et al., 2026; Fu et al., 2026; Gong et al., 2025; Wang et al., 2025c; Xu et al., 2026; Zhu et al., 2025). Fourth, oxygen- and redox-gas-regulating systems modify the interaction among oxidative stress, hypoxia, and NO, CO, or H2S signaling (Ding et al., 2022; Ding et al., 2020; Hao et al., 2022; Vong et al., 2018; Wang et al., 2026; Wang Y. et al., 2025; Wu C. R. et al., 2025). Fifth, biological redox-regulatory systems act primarily through endogenous antioxidant, mitochondrial, metabolic, inflammatory, or immunoregulatory pathways (Chen C. X. et al., 2025; Liu et al., 2016; Maranhao et al., 2017; Mesfin et al., 2025; Wang X. Y. et al., 2025; Xue et al., 2026).

These functions can coexist in one construct, but they are not mechanistically equivalent. Demonstration that treatment reduces ROS does not by itself establish ROS-responsive delivery. Strong evidence of responsiveness requires showing that oxidative conditions alter a measurable material property such as cleavage, degradation, release rate, or cargo availability, ideally relative to a matched low-ROS or non-responsive control.

#### Mechanistic hierarchy: From Material-ROS interaction to cardiac remodeling

The biological effects of these platforms can be arranged hierarchically. Primary mechanisms are directly produced by material chemistry or payload action, including ROS-triggered release, ROS consumption, catalytic conversion, antioxidant signaling, or targeted intracellular delivery. Proximal cellular consequences include mitochondrial stabilization, preservation of membrane potential, inhibition of mPTP opening, calcium regulation, restoration of mitochondrial metabolism, and suppression of apoptosis, ferroptosis, pyroptosis, or cuproptosis (Cheng et al., 2019; Hu et al., 2026; Ikeda et al., 2016; Wang et al., 2025c; Wang et al., 2026; Wang Y. et al., 2024). Inflammatory and immunometabolic changes may arise secondarily from reduced injury or directly through macrophage-, monocyte-, or immune-directed therapy (Hu et al., 2024; Liao et al., 2025; Pan et al., 2025; Xue et al., 2026). Angiogenesis, fibrosis, scar organization, ventricular geometry, and cardiac function are later tissue-level outcomes.

This hierarchy prevents mechanistic over-attribution. Mitochondria-targeted CsA nanoparticles reduced infarct size and improved 28-day remodeling without altering inflammatory monocyte recruitment (Ikeda et al., 2016), showing that benefit did not require suppression of that inflammatory compartment. Conversely, macrophage-directed systems can target inflammation directly rather than relying on inflammation to decline secondarily after ROS removal (Hu et al., 2024; Pan et al., 2025; Xue et al., 2026). The relationship among redox control, mitochondrial protection, inflammation, and remodeling is therefore platform dependent rather than universally linear.

Figure 3 summarizes this hierarchy from the primary material-redox interaction through proximal mitochondrial and cell-death responses to inflammatory or reparative effects and later ventricular remodeling, while depicting tissue-, cell-, and organelle-level targeting as an orthogonal precision-delivery layer.

**FIGURE 3:**
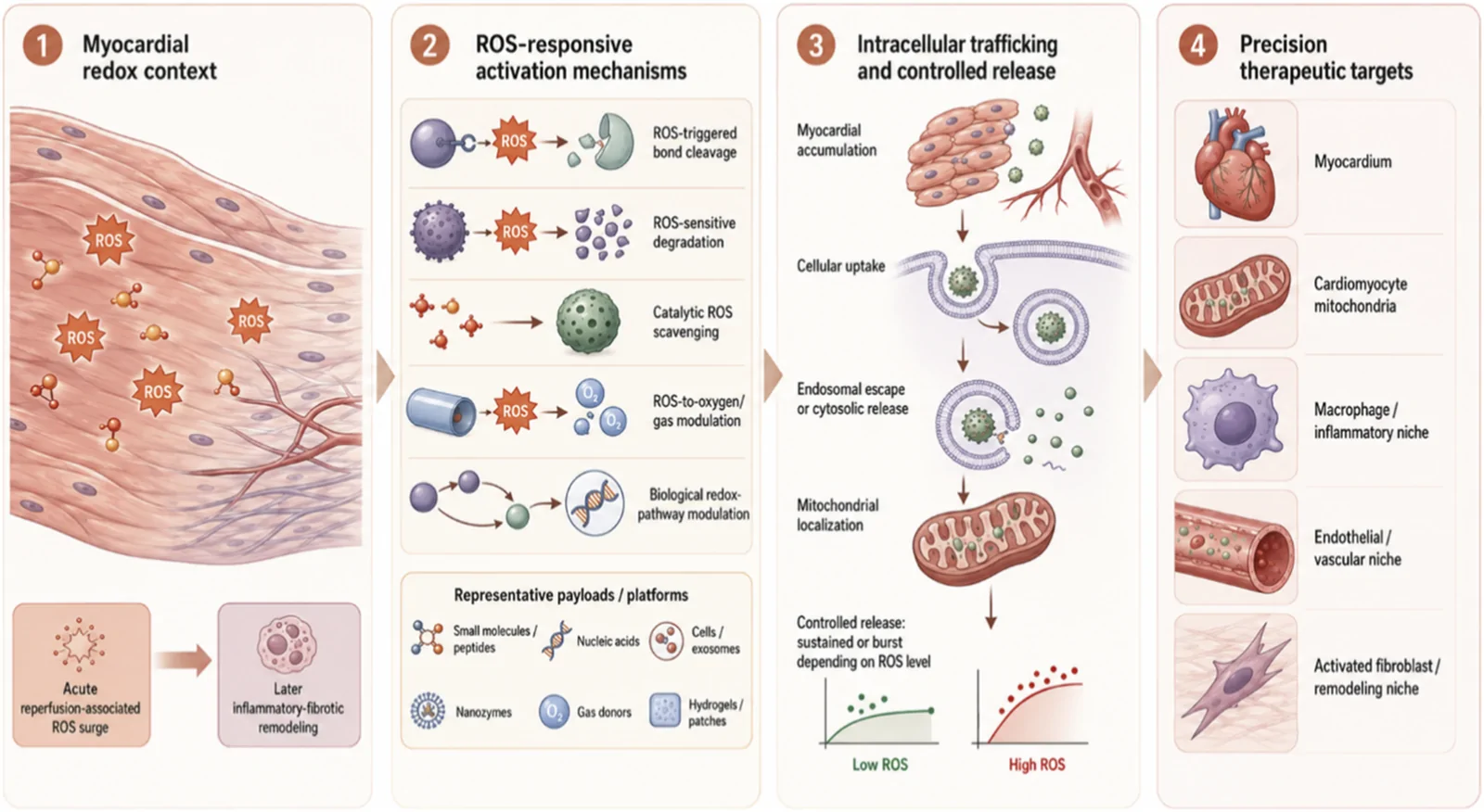
Hierarchical design and mechanism of ROS-interacting biomaterials in MIRI and post-infarction remodeling. The figure distinguishes the primary material or therapeutic interaction - ROS-triggered cleavage or release, direct scavenging, catalytic detoxification, oxygen or gas regulation, or biological redox-pathway modulation - from subsequent biological responses. Proximal responses include mitochondrial stabilization and regulation of apoptosis, ferroptosis, pyroptosis, or cuproptosis; inflammatory and immunometabolic changes may occur downstream or as independently targeted mechanisms; and angiogenesis, fibrosis, ventricular remodeling, and cardiac-function preservation represent later tissue-level outcomes. Tissue-, cell-, and organelle-targeting strategies are shown as an orthogonal precision layer (*Prepared with the assistance of Gemini NotebookLM pro and OpenAI ChatGPT Plus*).

#### ROS-triggered release and degradable systems

ROS-triggered systems provide the most direct realization of precision delivery because oxidative pathology itself governs material behavior. Thioketal-, boronic ester-, Schiff-base-, disulfide-, and related responsive chemistries have been used to control matrix degradation or therapeutic release (Cao et al., 2026; Hu et al., 2026; Qin et al., 2026; Song L. et al., 2025; Wang Y. et al., 2024; Zhen et al., 2025). PUE@TK/CHP-L combined a thioketal-responsive element with a cardiac homing peptide, enabling ischemic-myocardial targeting and drug release under elevated intracellular ROS (Wang Y. et al., 2024). OD@G4CAsi-FDX1 co-delivered caffeic acid and si-FDX1 within a ROS-responsive hydrogel to reduce oxidative stress and modulate cuproptosis (Hu et al., 2026). SaB/Cand core-shell microgels demonstrated H2O2-accelerated candesartan release, reaching 68% over 21 days compared with 24% in PBS (Song L. et al., 2025). Apelin-13@Gel TK released cargo more rapidly in the high-ROS injury core and more slowly in the lower-ROS border zone, linking spatial redox heterogeneity to early anti-apoptotic and later vascular and reparative effects (Zhen et al., 2025).

The major advantage of this class is the potential to couple therapy to the intensity, location, or temporal evolution of oxidative injury. The major limitation is inconsistent quantitative validation: only a subset of studies report trigger concentrations, release percentages, spatially differentiated release, or matched non-responsive controls. Sophisticated chemistry should therefore not be equated with superior biological precision unless responsiveness itself is demonstrated.

Redox responsiveness should also be specified precisely. For example, PF543 nanoparticles in study (Ji et al., 2023) were responsive to reduced glutathione and targeted activated cardiac fibroblasts. This is a redox-adaptive strategy but mechanistically distinct from ROS-triggered cleavage.

#### Direct ROS scavenging and antioxidant delivery

Direct scavenging platforms reduce oxidative burden or improve delivery of antioxidant therapeutics without requiring ROS-cleavable release. Examples include bilirubin nanoparticles (Ai et al., 2021), mitochondria-targeted resveratrol nanoparticles (Cheng et al., 2019), macrophage-selective quercetin nanomedicine (Hu et al., 2024), an ROS-scavenging ISMN-liposome composite hydrogel (Hua et al., 2025), Cur-PEG-GQDs (Rostamzadeh et al., 2023), antioxidant hydrogels (Liao et al., 2022; Wang et al., 2022), and MitoQ-containing local delivery systems (Tan et al., 2024). Their strength is that activity does not depend on reaching a precise ROS threshold for carrier cleavage. Targeting can instead be achieved through myocardial localization, mitochondrial delivery, immune-cell selectivity, biomimetic trafficking, or local retention.

MCTD-NPs increased resveratrol distribution within ischemic myocardium and mitochondria, reduced mitochondrial ROS and mPTP opening, and decreased infarct size (Cheng et al., 2019). Que@MOF/Man preferentially entered macrophages rather than cardiomyocytes and distributed within injured myocardium, connecting antioxidant therapy with macrophage reprogramming (Hu et al., 2024). In rat MI, the ISMN-liposome composite hydrogel improved cardiac function, reduced infarct size, and increased left ventricular wall thickness (Hua et al., 2025), whereas Cur-PEG-GQDs improved hemodynamic and cardiac functional indices while reducing infarct size, fibrosis, and oxidative imbalance after LAD ligation (Rostamzadeh et al., 2023). These studies provide strong evidence for redox-directed therapy but weaker evidence for stimulus-controlled delivery unless a separate responsive element is demonstrated.

#### Catalytic nanozymes and enzyme-based detoxification

Catalytic nanozymes represent a distinct strategy because their proposed advantage is repeated or sustained conversion of ROS rather than stoichiometric consumption of an antioxidant molecule. The evidence includes encapsulated SOD (Altshuler et al., 2021), ceria-based nanozymes (Chen X. R. et al., 2025; Dai et al., 2026; Wang et al., 2025c; Zhu et al., 2025; Wang J. H. et al., 2025), Fe-, Cu-, and Mn-single-atom nanozymes (Fu et al., 2026), PtIr bimetallic nanozymes (Gong et al., 2025), and ZnSrMo-LDH/Cu systems with cascade antioxidant activity (Xu et al., 2026).

Fe-, Cu-, and Mn-single-atom nanozymes displayed complementary SOD-, catalase-, and peroxidase-like activities (Fu et al., 2026), while PtIr nanozymes combined SOD- and catalase-like activity with early reductions in oxidative injury and inflammation and later reductions in infarct size and fibrosis (Gong et al., 2025). Ce-Lut@TA increased cardiac accumulation, reduced oxidative and inflammatory injury, promoted M2 macrophage polarization, decreased infarct area, and improved cardiac function in a mouse MI model, with effects associated with MAPK signaling (Wang J. H. et al., 2025). ZnSrMo-LDH/Cu-BSA showed ROS-scavenging capacity reported as 2.97-fold greater than Fe3O4 and also promoted vascular regeneration (Xu et al., 2026). More complex systems such as B-Ce@D-P@M and D/Ce@mPDA-C/P combined catalytic scavenging with mitochondrial targeting, calcium or iron regulation, and ferroptosis control (Wang et al., 2025c; Zhu et al., 2025).

The mechanistic strength of catalytic systems is potential durability. The corresponding limitation is that multifunctional constructs complicate attribution of benefit, and inorganic persistence becomes a safety question. Metal- and inorganic-containing nanozymes require specific evaluation of particle transformation, ion release, chronic deposition, and clearance rather than assuming that catalytic persistence is uniformly advantageous.

#### Mitochondrial targeting as an orthogonal precision strategy

Mitochondrial targeting is not a separate ROS-interaction mechanism; it is a precision layer that can be superimposed on scavengers, responsive carriers, nanozymes, gases, or conventional therapeutics. This strategy is especially coherent in MIRI because mitochondria are closely linked to reperfusion-associated ROS generation, mPTP opening, calcium dysregulation, membrane failure, and regulated cell death.

CsA-loaded PLGA nanoparticles localized to mitochondria in ischemic myocardium, reduced infarct size at a lower CsA concentration than free CsA, and improved 28-day remodeling (Ikeda et al., 2016). MCTD-NPs delivered resveratrol to mitochondria and reduced mitochondrial ROS and mPTP opening (Cheng et al., 2019). B-Ce@D-P@M combined mitochondrial targeting with ROS scavenging and calcium chelation (Wang et al., 2025c), while EM@CO used sequential myocardial and mitochondrial targeting to reduce mitochondrial ROS generation, mitochondrial DNA release, and pyroptosis (Wang et al., 2026). SS31-modified nanomicelles within a responsive hydrogel similarly coupled local release with mitochondria-directed antioxidant activity (Zhang et al., 2022). No quantitative head-to-head comparison among mitochondrial targeting architectures was identified, so relative superiority cannot be assigned.

#### Oxygen generation and redox-gas regulation

Oxygen-generating systems address the combined hypoxic and oxidative environment of infarcted myocardium. RCGel combined ROS scavenging with oxygen generation and improved mesenchymal stem-cell retention, angiogenesis, fibrosis, and cardiac function (Ding et al., 2022). A catalase-containing ROS-cleavable hydrogel converted H2O2 to oxygen and water and crosslinked within 3 s (Ding et al., 2020). Additional oxygen-releasing constructs included a calcium-peroxide cardiac patch and a self-oxygenating regenerative hydrogel (Shiekh et al., 2022; Song Z. et al., 2025). These designs may be especially relevant to regenerative MI repair, although optimization of oxygen generation relative to tissue demand remains incompletely standardized.

NO-, CO-, and H2S-based systems extend redox therapy beyond antioxidant scavenging. CS-B-NO released NO in response to ROS and was compared with an NO-only hydrogel (Hao et al., 2022). NO-RIG combined ROS scavenging with sustained NO release and outperformed simpler NO-releasing or ROS-scavenging control gels for infarct and functional outcomes (Vong et al., 2018). EM@CO provided targeted mitochondrial CO delivery (Wang et al., 2026), Rh-PEG-CO enabled ultrasound-controlled CO release (Wu C. R. et al., 2025), and ZnS/SOD microneedles combined enzymatic ROS control with acidic-pH-responsive H2S generation (Wang Y. et al., 2025). These systems are mechanistically powerful but require particularly careful control of dose, systemic exposure, and hemodynamic effects.

#### Biological redox regulation and immunometabolic therapy

Several interventions target signaling networks that generate, amplify, or respond to oxidative stress rather than relying primarily on ROS-cleavable material behavior. Selenium nanoparticles disrupted a STAT1-ROS cycle and were associated with preserved mitochondrial respiration, reduced apoptosis, reduced macrophage infiltration, and improved cardiac function (Chen C. X. et al., 2025). Keap1-inhibitory protein-like polymers activated endogenous Nrf2 signaling (Mesfin et al., 2025), while MitoQ@MMNv combined cardiomyocyte ROS reduction with restoration of oxidative phosphorylation in inflammatory macrophages (Xue et al., 2026). Acacetin phosphate preserved endogenous SOD-2 and thioredoxin during I/R (Liu et al., 2016), and LDE-MTX increased antioxidant enzymes and adenosine bioavailability after MI (Maranhao et al., 2017).

These systems illustrate why inflammation should not automatically be framed as downstream of ROS suppression. Macrophage metabolism, efferocytosis, endogenous antioxidant signaling, and fibroblast activation can themselves be primary therapeutic targets (Hu et al., 2024; Mesfin et al., 2025; Pan et al., 2025; Xue et al., 2026). In multifunctional systems, reductions in ROS may therefore be both a cause and a consequence of broader biological regulation.

#### Targeting architecture

Targeting should be evaluated independently from ROS-interaction mechanism. Cardiac homing peptides, platelet membranes, macrophage membranes, mannan, mitochondrial ligands, SS31, TPP modification, TOM20/VDAC affinity, and fibroblast-directed approaches provide tissue-, cell-, or organelle-level specificity (Hu et al., 2024; Ji et al., 2023; Li et al., 2026; Wang et al., 2025c; Wang et al., 2026; Wang X. Y. et al., 2025; Wang Y. et al., 2024; Zhang et al., 2022; Zhu et al., 2025). A platform can be highly ROS-responsive but weakly targeted, or strongly targeted without using ROS to control release. PDMC/CHP@S/V combined cardiac-homing-peptide targeting with a macrophage-evasive phosphorylcholine-containing shell and antioxidant catechol groups; in AMI models it reduced ROS and apoptosis, restored mitochondrial membrane potential, and improved cardiac function, fibrosis, and ventricular remodeling (Yang et al., 2026). The most persuasive precision-delivery systems combine targeting and redox control, but each added ligand, coating, membrane, or targeting component also increases formulation and manufacturing complexity.

#### Administration route and timing across acute reperfusion injury and post-infarction remodeling

Administration route is a determinant of therapeutic performance rather than a purely technical feature. Local and systemic strategies address different delivery constraints and should be compared in relation to disease stage, treatment window, and required duration of exposure.

Local myocardial administration was used extensively for hydrogels, microgels, nanozymes, and other systems intended to maximize retention within injured tissue. Intramyocardial injection was reported for NP-SOD, single-atom nanozymes, OD@G4CAsi-FDX1, PEG-b-PPS nanoparticles, PCB2 hydrogel, MitoQ hydrogel, and R gel (Altshuler et al., 2021; Fu et al., 2026; Hu et al., 2026; Li et al., 2020; Qin et al., 2026; Tan et al., 2024; Wang et al., 2022). NO-RIG was administered intracardially (Vong et al., 2018), while patch or myocardial-surface application was used in several regenerative and remodeling-oriented interventions (Shiekh et al., 2022; Sun et al., 2024; Tan et al., 2024; Yang J. Q. et al., 2025). Local administration can increase myocardial exposure and support prolonged or staged release, but its invasiveness may limit routine use unless delivery can be integrated into a clinically acceptable catheter-based or surgical workflow.

Route itself can change efficacy. In a direct comparison using the same MitoQ-containing hydrogel, both myocardial-surface patches and intramyocardial injection produced benefit, but intramyocardial administration provided superior improvement in cardiac structure and function during equivalent treatment cycles (Tan et al., 2024). This finding shows that apparent platform performance can depend on delivery geometry independently of the payload.

Systemic delivery may be more compatible with narrow peri-reperfusion windows because it avoids direct myocardial instrumentation. Intravenous administration was reported for several targeted or redox-modulating systems (Chen C. X. et al., 2025; Ikeda et al., 2016; Liu et al., 2016; Mesfin et al., 2025; Wang et al., 2026; Weng et al., 2022; Yang W. L. et al., 2025), while tail-vein dosing was used in additional nanoparticle studies (Huang et al., 2022; Liao et al., 2025; Shi et al., 2024). These approaches depend on circulation, vascular access to injured tissue, targeting efficiency, off-target sequestration, and predictable clearance.

Treatment timing further separates acute MIRI from later repair. CsA nanoparticles were administered at reperfusion onset (Ikeda et al., 2016), whereas TPE-ss COF@Matrine was administered shortly after a defined ischemic interval (Huang et al., 2022). By contrast, lutein@DTPP was given for 3 days before MIRI (Shi et al., 2024), making it mechanistically informative but less directly applicable to unplanned reperfusion therapy. Several local systems were explicitly programmed for sequential repair: S1&FT/Lipo-QCFT coupled rapid early release of S1QEL1.1 and tannic acid with later FT011 delivery to address ROS/inflammation before fibrosis (Luo Q. et al., 2025); a PVA-dopamine/N(BA)3 hydrogel combined rapidly released anti-inflammatory/pro-angiogenic nanoparticles with longer-term galunisertib delivery (Wu Y. et al., 2025); and ALG/Zn/HA-QCT used early Zn/HA-mediated ROS scavenging followed by quercitrin-mediated suppression of fibroblast activation and collagen formation (Zhai et al., 2025). These stage-programmed designs are more logically aligned with evolving post-infarction repair than with a single narrow reperfusion window.

The design implication is stage dependent: acute MIRI favors rapid availability and clinically feasible peri-reperfusion delivery, whereas post-MI repair can justify longer local residence and staged release. Neither local nor systemic administration can be considered categorically superior; the relevant question is whether the route provides adequate myocardial exposure at the required stage without disproportionate procedural, pharmacokinetic, or safety burden.

#### Comparative interpretation and clinical applicability

No single platform class can presently be identified as superior across MIRI and post-infarction remodeling. ROS-triggered hydrogels and microgels provide a strong rationale for local retention, staged release, and spatial control, but their frequent reliance on direct cardiac administration creates an implementation barrier. Systemically administered nanoparticles and liposomes offer greater potential for peri-reperfusion treatment and multilevel targeting, but their translational value depends on stronger quantitative biodistribution and clearance data. Catalytic nanozymes may provide sustained redox regulation but raise persistence and long-term safety concerns. Direct antioxidant and biological redox-modulating platforms can be mechanistically simpler and may avoid the need for a calibrated activation threshold but generally provide less evidence that treatment availability is controlled by the injured microenvironment. Gas- and oxygen-regulating systems address physiologically important pathways beyond ROS scavenging but require particularly precise exposure control.

The most clinically credible strategy may therefore be the minimum-complexity platform that delivers sufficient therapeutic material to the appropriate myocardial compartment at the appropriate disease stage with measurable added benefit over a simpler comparator. Direct head-to-head studies comparing major platform classes under the same injury model, treatment window, payload, and outcome framework were not identified. Current evidence supports comparative design principles rather than a definitive ranking of hydrogels, nanoparticles, nanozymes, or other ROS-interacting systems. Table 3 summarizes the comparative design characteristics, relative advantages, principal limitations, and most plausible contexts of the major ROS-interacting cardiovascular delivery strategies.

**TABLE 3:** Comparative design characteristics of ROS-interacting cardiovascular delivery platforms.

Platform strategy	Primary ROS/redox function	Relative advantage	Principal limitation	Most plausible context	Representative refs
ROS-triggered hydrogels/microgels	ROS-dependent cleavage, degradation, or staged release	High local retention; spatial and temporal control	Often requires direct cardiac administration; responsiveness not always quantified	Post-MI repair, fibrosis, staged remodeling; selected MIRI	(Cao et al., 2026; Hu et al., 2026; Qin et al., 2026; Song L. et al., 2025; Wang et al., 2022; Zhen et al., 2025)
ROS-responsive nanoparticles/liposomes	Triggered release plus systemic or cellular targeting	Potential peri-reperfusion use and multilevel targeting	Biodistribution, clearance, and activation thresholds inconsistently characterized	Acute MIRI and targeted cardiomyocyte delivery	(Li et al., 2020; Wang et al., 2024c; Weng et al., 2022)
Direct antioxidant/scavenging carriers	Direct reduction of oxidative burden	Mechanistically straightforward; compatible with targeting	Does not necessarily provide stimulus-controlled release	Acute cytoprotection and microenvironment conditioning	(Ai et al., 2021; Cheng et al., 2019; Hu et al., 2024; Liao et al., 2022; Tan et al., 2024)
Catalytic nanozymes/enzyme carriers	Repeated catalytic ROS conversion	Potential sustained detoxification and multi-enzyme activity	Persistence, clearance, component attribution, chronic safety	MIRI, mitochondrial injury, persistent oxidative stress	(Altshuler et al., 2021; Chen et al., 2025b; Dai et al., 2026; Fu et al., 2026; Gong et al., 2025; Wang et al., 2025c; Xu et al., 2026; Zhu et al., 2025)
Mitochondria-targeted systems	Delivers scavengers, inhibitors, nanozymes, or gases to mitochondria	Strong alignment with mitochondrial ROS and regulated cell death	Added targeting complexity; no head-to-head architecture comparison	Particularly relevant to acute MIRI	(Cheng et al., 2019; Ikeda et al., 2016; Wang et al., 2025c; Wang et al., 2026; Wang et al., 2025d; Zhang et al., 2022; Zhu et al., 2025)
Oxygen-generating systems	Couples redox regulation with local oxygen production	Addresses hypoxia and oxidative stress together	Oxygen-generation requirements and exposure not standardized	Regenerative MI repair and cell-support strategies	(Ding et al., 2022; Ding et al., 2020; Shiekh et al., 2022; Song Z et al., 2025)
NO/CO/H_2_S systems	Modulates redox-gas signaling with local or triggered release	Targets signaling pathways beyond ROS scavenging	Dose-release control and systemic exposure require dedicated safety study	MIRI, angiogenesis, inflammation, pyroptosis, remodeling	(Hao et al., 2022; Vong et al., 2018; Wang et al., 2026; Wang et al., 2025e; Wu et al., 2025a)
Biological redox/immunometabolic systems	Modulates endogenous antioxidant, metabolic, or inflammatory circuitry	Targets self-amplifying biological pathways	Causal attribution between ROS and broader biology can be difficult	Inflammation resolution and post-MI repair	(Chen et al., 2025a; Hu et al., 2024; Mesfin et al., 2025; Pan et al., 2025; Wang et al., 2025d; Xue et al., 2026)

### Quantitative validation of responsiveness, delivery, and therapeutic performance

#### Quantitative evidence as an evidence chain

The quantitative evidence is heterogeneous in both depth and purpose. Some studies provide detailed physicochemical characterization but limited pharmacokinetic information; others demonstrate myocardial targeting or stimulus-dependent release without comprehensive material characterization; and many report cardiac functional improvement without quantitatively linking that benefit to responsive material behavior. Numerical therapeutic outcomes alone should therefore not be used to rank platforms.

A more rigorous assessment requires an evidence chain linking material properties, stimulus-dependent or redox-active behavior, delivery or localization, and cardiac therapeutic outcomes. The strongest evidence is obtained when these levels are demonstrated within the same platform and compared with an appropriate non-responsive, free-drug, carrier, or untargeted control. Particle size, drug-loading efficiency, ROS-scavenging capacity, myocardial fluorescence intensity, LVEF, fibrosis, and survival answer different questions and should be interpreted at their respective evidentiary levels.

#### Material characterization establishes formulation quality, not therapeutic superiority

Selected platforms reported quantitative physicochemical characteristics. BN-PEG-NLC measured 83.9 nm, had a zeta potential of −32.1 mV, and an entrapment efficiency of 83.5% (Zhang et al., 2016). Curcumin-gold nanoparticles measured 32±9 nm with a zeta potential of −29.6 mV, while the associated hydrogel showed 70%–80% interconnected porosity (Zhao et al., 2026). D/Ce@mPDA-C/P incorporated 2–3 nm CeO2 particles within composite nanoparticles of approximately 130 nm (Zhu et al., 2025). Other material metrics included approximately 220-μm Treg-regulating microgels (Wang S. Q. et al., 2024), a catalase-containing hydrogel that crosslinked within 3 s (Ding et al., 2020), and NO-RIG retention in myocardial tissue for more than 10 days (Vong et al., 2018).

These measurements establish formulation properties relevant to loading, injectability, stability, or residence. They do not establish therapeutic superiority. A smaller particle, higher loading efficiency, greater porosity, or faster gelation is clinically meaningful only if it improves administration, localization, release, efficacy, safety, or manufacturability.

#### Quantitative ROS responsiveness

The clearest distinction in this literature is between systems that demonstrate ROS-dependent material behavior and systems described as ROS-responsive mainly because they reduce oxidative stress. SaB/Cand microgels provide one of the strongest quantitative examples: candesartan release reached 68% over 21 days in the presence of H2O2 compared with 24% in PBS (Song L. et al., 2025). Apelin-13@Gel TK provides a complementary spatial example, with more rapid release in the high-ROS injury core and slower release in the lower-ROS border zone (Zhen et al., 2025). These studies show that pathological redox conditions can function as delivery cues rather than merely therapeutic targets.

Several other systems were described as ROS-sensitive or redox-responsive without comparable trigger concentrations, release percentages, activation thresholds, or matched low-ROS release conditions in the extracted evidence (Cao et al., 2026; Hao et al., 2022; Hu et al., 2026; Qin et al., 2026; Shi et al., 2024; Wang Y. et al., 2024; Zhang et al., 2022). These remain mechanistically relevant, but the quantitative strength of the responsiveness claim is not uniform. BN-PEG-NLC, by contrast, demonstrated burst followed by prolonged release and major pharmacokinetic enhancement (Zhang et al., 2016), but the evidence does not identify ROS as the trigger governing release; it is therefore a delivery benchmark rather than an example of ROS-triggered release.

#### Quantitative delivery and targeting

Precision delivery is most convincing when localization is measured relative to an appropriate comparator rather than inferred from the presence of a targeting ligand. HI@PSeP-IMTP increased maximal fluorescence and photoacoustic signals within the MIRI region by approximately 32% and 40%, respectively, relative to the non-IMTP formulation (Ma X. B. et al., 2025). PUE@CHP/PM-L increased myocardial retention and cardiomyocyte uptake while decreasing hepatic and splenic sequestration (Li et al., 2026). Que@MOF/Man showed macrophage-selective uptake and distribution within injured myocardium (Hu et al., 2024), and B-Ce@D-P@M incorporated fluorescence and PET/CT assessment of myocardial targeting (Wang et al., 2025c).

BN-PEG-NLC provided one of the more detailed pharmacokinetic datasets, increasing baicalin area under the curve 7.2-fold relative to baicalin solution and 3-fold relative to non-PEGylated lipid carriers, with higher drug concentrations in the heart (Zhang et al., 2016). These data demonstrate formulation-associated exposure gains but should not be directly compared with percentage changes in fluorescence, retention, or cellular uptake from other studies because the endpoints are different.

#### Therapeutic numbers are most reliable as within-study comparisons

Several studies report substantial improvements in cardiac outcomes, but the appropriate unit of interpretation is generally the within-study treatment-versus-control comparison. NP-TPI1/P produced an LVEF of 49.42±1.88% compared with 31.61±2.30% in PBS-treated mice at day 21 after AMI, while fibrotic area was 11.60±1.60% versus 25.48±1.98% (Pan et al., 2025). EGCG@MPDA/CS produced an LVEF of 51.25±1.73% compared with 29.31±0.78% in PBS-treated rats at 28 days (Wang T. H. et al., 2024). SMM@Gel produced an LVEF of 52.3±4.1% compared with 38.5±3.2% in saline-treated animals and reduced infarct size from 32.1±3.8% to 18.7±2.5% (Yang J. Q. et al., 2025).

Although the treated LVEF values appear numerically similar, they should not be interpreted as evidence of equivalent or ranked efficacy because the studies used different disease models, routes, controls, treatment schedules, and time points. The same caution applies to fibrosis. Rb1/PDA-hydrogel reduced fibrotic area from 58.4% to 5.5% at 28 days (Zheng et al., 2024), but fibrosis definitions, anatomical denominators, and comparator conditions vary across studies. The numerically largest change cannot be assumed to identify the most effective platform.

#### Comparator structure strengthens quantitative interpretation

Quantitative efficacy becomes more informative when comparator groups permit attribution of benefit to formulation or material design. LDE-MTX was compared with both empty LDE and commercial methotrexate after MI (Maranhao et al., 2017); the nanoparticle formulation produced a reported 40% improvement in left ventricular systolic function and broader structural and biochemical benefits, whereas commercial methotrexate produced only minor effects and did not improve cardiac function or infarct size. R gel was compared with a non-reactive hydrogel at 3 days and 8 weeks (Wang et al., 2022), NO-RIG with simpler NO-releasing and ROS-scavenging gels (Vong et al., 2018), and MitoQ hydrogel by both myocardial surface application and intramyocardial injection (Tan et al., 2024). These designs are especially valuable because one material or delivery feature is altered while other experimental conditions remain similar.

#### Catalytic activity requires the same caution

Catalytic measurements can appear particularly suitable for ranking nanozymes, but assay conditions and *in vivo* delivery differ. ZnSrMo-LDH/Cu-BSA exhibited ROS-scavenging capacity 2.97-fold greater than Fe3O4 and reduced cardiomyocyte apoptosis to 9.4% *in vitro* and 20.7% *in vivo* of corresponding MI/R-group levels (Xu et al., 2026). This is a meaningful within-study catalytic comparison. Fe-, Cu-, and Mn-single-atom nanozymes and PtIr nanozymes were also associated with enzyme-mimetic activity and improved cardiac outcomes (Fu et al., 2026; Gong et al., 2025). These findings support catalytic redox regulation but do not establish that the platform with the largest *in vitro* scavenging value will produce the greatest myocardial benefit or the best safety profile.

#### Large-animal and regenerative outcomes

The miR-19a/b ROS-eliminating hydrogel provides the most advanced large-animal quantitative signal identified. Approximately 20.47% of adult cardiomyocytes at injected sites underwent cell division in the mouse experiments, while the porcine MI study reported a decrease in infarct size from 40% to 18%, improved cardiac function and muscle mass, and survival of all treated pigs to scheduled heart harvest at day 50 (Wang et al., 2025b). These results merit emphasis because large-animal validation is rare, but they remain evidence for one platform rather than proof that ROS-responsive hydrogels as a class are clinically ready.

#### Comparative interpretation of quantitative evidence

Taken together, therapeutic efficacy is quantified more frequently than ROS responsiveness itself. Precision delivery is increasingly measurable through imaging, retention, cell selectivity, mitochondrial localization, and pharmacokinetic enhancement (Hu et al., 2024; Li et al., 2026; Ma X. B. et al., 2025; Wang et al., 2025c; Zhang et al., 2016), but these endpoints are not standardized. The strongest platforms are not necessarily those reporting the largest numerical therapeutic effect, but those providing the clearest causal chain from material property to responsive behavior or targeting, then to biological mechanism and cardiac benefit. Future studies should report physiologically relevant trigger concentrations, matched responsive and non-responsive release kinetics, whole-organ biodistribution and clearance, appropriate carrier and free-drug controls, and standardized acute and remodeling endpoints. Table 4 summarizes the key quantitative evidence domains, representative findings, and interpretive limits, while Figure 4 presents the evidence chain from material characterization through responsive behavior and precision delivery to cardiac outcome.

**TABLE 4:** Quantitative evidence and appropriate interpretation of ROS-interacting cardiovascular biomaterials.

Evidence domain	Representative finding	What it supports	What it does not establish	Refs
Physicochemical formulation	BN-PEG-NLC: 83.9 nm, −32.1 mV, 83.5% entrapment; D/Ce@mPDA-C/P: 2–3 nm CeO_2_ within ∼130 nm composites	Defined formulation properties	Therapeutic superiority	(Zhang et al., 2016; Zhu et al., 2025)
ROS-dependent release	SaB/Cand: 68% candesartan release over 21 days in H_2_O_2_ vs. 24% in PBS	Direct evidence that oxidative conditions alter release	General superiority over other responsive chemistries	(Song L. et al., 2025)
Spatial ROS-responsive release	Faster Apelin-13 release in high-ROS core; slower release in lower-ROS border zone	Spatial coupling of release to myocardial ROS heterogeneity	Universal performance across infarct models	(Zhen et al., 2025)
Quantified targeting	HI@PSeP-IMTP: ∼32% greater fluorescence and ∼40% greater photoacoustic signal than non-IMTP formulation	Added localization from targeting architecture	Complete biodistribution or clearance	(Ma et al., 2025b)
Pharmacokinetic enhancement	BN-PEG-NLC: 7.2-fold AUC vs. solution and 3-fold vs. non-PEG carrier	Formulation-associated exposure and cardiac-delivery advantage	ROS-triggered release	(Zhang et al., 2016)
Matched formulation comparison	R gel vs. non-reactive hydrogel at 3 days and 8 weeks	Stronger attribution to responsive material behavior	Class-wide hydrogel superiority	(Wang et al., 2022)
Within-study cardiac function	NP-TPI1/P: LVEF 49.42±1.88% vs. 31.61±2.30% PBS	Therapeutic benefit within that AMI model	Superiority over other studies with different models	(Pan et al., 2025)
Catalytic comparison	ZnSrMo-LDH/Cu-BSA: 2.97-fold ROS-scavenging capacity vs. Fe3O4	Greater catalytic performance under reported assay conditions	Greater clinical efficacy or safety than other nanozymes	(Xu et al., 2026)
Large-animal signal	miR-19a/b hydrogel: porcine infarct size 40%–18%; all treated pigs survived to scheduled day-50 harvest	Advanced platform-specific preclinical efficacy	Clinical readiness or class-wide efficacy	(Wang et al., 2025b)

**FIGURE 4:**
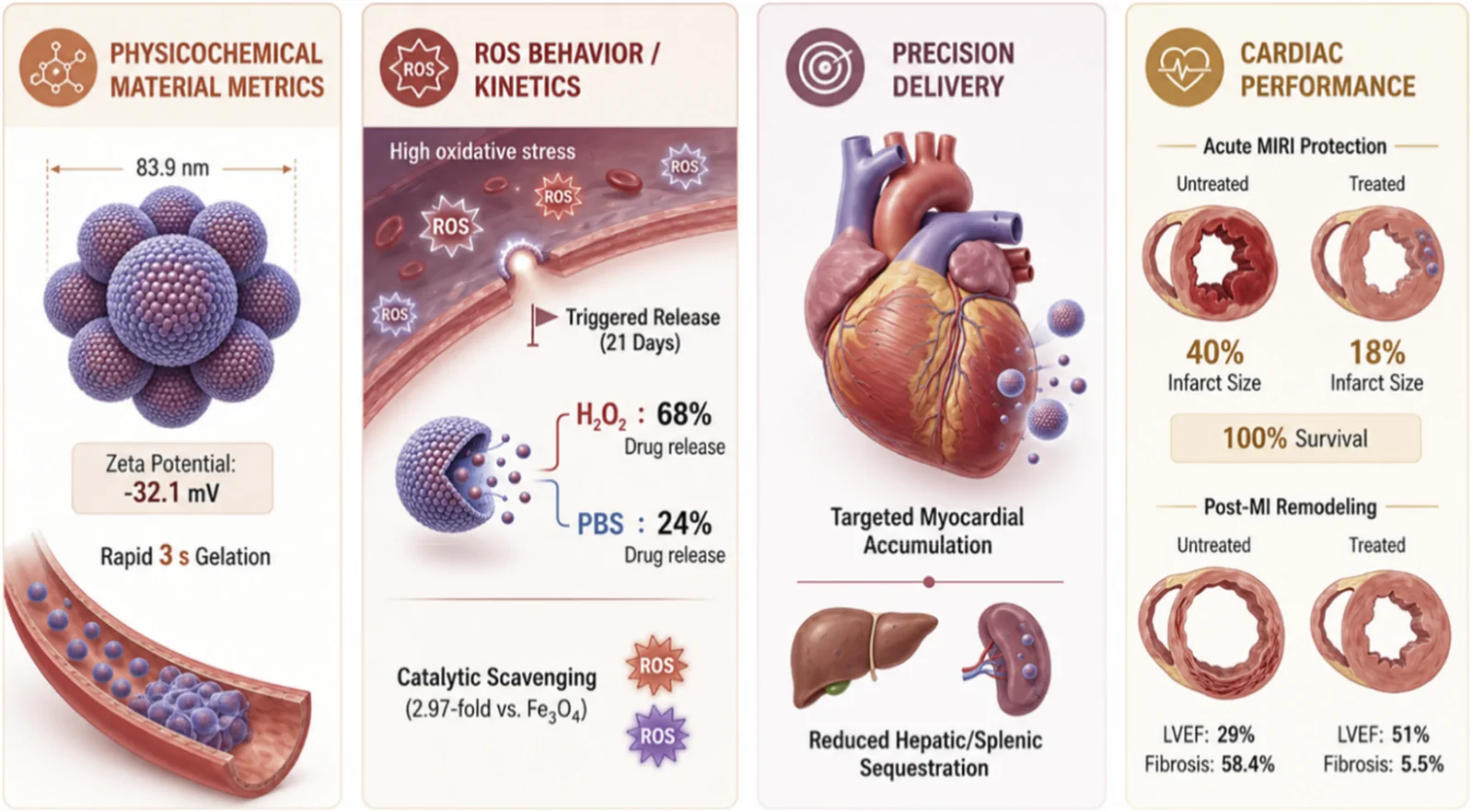
Evidence chain for quantitative validation of ROS-interacting cardiovascular biomaterials. Quantitative evidence should be interpreted sequentially from material characterization to demonstrated responsive behavior, precision delivery, biological action, and cardiac outcome. Particle size, loading, gelation, or catalytic activity establish material performance; ROS-dependent release or degradation establishes stimulus responsiveness; biodistribution, imaging, retention, cellular uptake, or mitochondrial localization establish delivery; and infarct size, LVEF, fibrosis, remodeling, rhythm outcomes, or survival establish therapeutic performance. The strongest evidence connects several levels within the same platform and includes appropriate non-responsive, untargeted, carrier, or free-drug controls (*Prepared with the assistance of Gemini NotebookLM pro and OpenAI ChatGPT Plus*).

### Safety, limitations, and translational readiness

#### Translational readiness lags behind therapeutic sophistication

The reviewed platforms demonstrate increasingly sophisticated control over myocardial localization, intracellular targeting, ROS-dependent release, catalytic redox regulation, and post-infarction repair. Their translational evidence is less mature. Most studies remain based on *in vitro* assays and rodent cardiovascular models, with selected pharmacokinetic, biodistribution, electrophysiological, or longer-term assessments and only one explicitly identified porcine MI study (Ai et al., 2021; Li et al., 2026; Ma X. B. et al., 2025; Wang et al., 2025c; Zhang et al., 2016; Wang et al., 2025b). No human observational or clinical-trial evidence was identified.

Therapeutic efficacy and translational readiness answer different questions. Reduction of infarct size, fibrosis, ROS, or inflammatory signaling does not establish whether a material can be administered reproducibly, remain within an acceptable exposure range, avoid delayed cardiovascular toxicity, undergo predictable degradation or clearance, and be manufactured consistently. Likewise, myocardial accumulation or stimulus-responsive release does not independently establish systemic safety.

#### Preliminary tolerability is not equivalent to comprehensive safety

Several studies provide encouraging but limited tolerability signals. Fe-, Cu-, and Mn-single-atom nanozymes were reported to reduce myocardial injury without systemic toxicity in a rat MI model (Fu et al., 2026). Fibroblast-targeted PF543 nanoparticles produced lower systemic toxicity than conventional systemic drug administration (Ji et al., 2023). BRAP caused no identified hepatic or renal functional abnormalities during 7 days of daily high-dose administration in mouse I/R experiments (Lee et al., 2015). Selected cross-organ studies similarly reported absence of obvious toxicity or favorable biocompatibility (Feng et al., 2022; Zhang et al., 2026). These observations address different and limited safety questions and should not be combined into a general conclusion that ROS-responsive biomaterials are safe.

Standardized evaluation of hematology, hemolysis, coagulation, complement activation, immunogenicity, cytokine-release responses, repeated-dose tolerability, comprehensive major-organ histology, reproductive toxicity, degradation-product toxicity, and chronic tissue accumulation was not consistently identified. The appropriate conclusion is that selected platforms show preliminary tolerability while comprehensive platform-specific safety remains insufficiently characterized.

#### Cardiovascular safety requires greater priority

Cardiovascular biomaterials require safety assessment beyond conventional organ toxicology because treatment is delivered to or accumulates within electrically and mechanically vulnerable myocardium. A small number of studies reported electrophysiologically relevant outcomes. Curcumin nanoparticles reduced QRS enlargement and QT/QTc abnormalities in an isoproterenol-associated myocardial-injury model (Boarescu et al., 2019). Intravenous acacetin phosphate reduced ventricular-arrhythmia score, arrhythmia duration, and ventricular fibrillation in rat myocardial I/R (Liu et al., 2016). The conductive FLCM hydrogel preserved connexin 43 and reduced ventricular-arrhythmia incidence following MI (Taiwaikuli et al., 2026). R&C-Gel also enhanced rhythmic intracellular Ca2+ activity and Cx43 expression while supporting cardiomyocyte engraftment and later functional repair (Zhan et al., 2022).

These findings are encouraging but are primarily therapeutic electrophysiological or electrical-integration outcomes rather than formal proarrhythmic safety studies. Systematic QT/QTc analysis, chronic rhythm monitoring, programmed electrophysiological testing, electrical mapping, and delayed-arrhythmogenicity assessment were not identified for most platforms. Cardiac safety should therefore be treated as a core translational endpoint rather than inferred from improved ventricular function or electrical-coupling markers.

#### Targeting evidence is stronger than complete biodistribution evidence

Precision targeting is one of the strongest features of the field. Cardiac homing peptides, platelet and macrophage membranes, mitochondrial ligands, inflammatory-cell tropism, and related strategies have produced preferential myocardial accumulation, cellular uptake, or subcellular localization (Hu et al., 2024; Li et al., 2026; Ma X. B. et al., 2025; Pan et al., 2025; Wang et al., 2025c; Wang Y. et al., 2024; Xue et al., 2026; Zhu et al., 2025). HI@PSeP-IMTP increased maximal fluorescence and photoacoustic signals in the MIRI region by approximately 32% and 40% relative to its non-IMTP comparator (Ma X. B. et al., 2025). PUE@CHP/PM-L increased myocardial targeting and retention while decreasing hepatic and splenic sequestration (Li et al., 2026). B-Ce@D-P@M used fluorescence and PET/CT imaging to evaluate myocardial targeting (Wang et al., 2025c), and BN-PEG-NLC increased baicalin AUC 7.2-fold relative to solution and 3-fold relative to non-PEGylated carriers (Zhang et al., 2016).

Preferential accumulation should not be equated with a complete pharmacokinetic profile. Circulation half-life, dose proportionality, absolute organ exposure, target-to-nontarget ratios, excretion fractions, carrier-versus-payload disposition, and quantitative clearance pathways were inconsistently reported. EA-AuNPs were described as showing favorable excretion-based metabolism without overburdening other organs, but the available evidence did not specify the exact clearance route or quantitative extent (Yu et al., 2024). A platform can therefore show favorable targeting or qualitative disposition while still lacking the complete pharmacokinetic and clearance dataset needed for translation.

#### Local retention, degradation, and persistence

Injectable hydrogels, patches, and myocardial depots can overcome rapid washout and low systemic cardiac exposure. NP-SOD demonstrated enhanced intramyocardial retention (Altshuler et al., 2021), NO-RIG remained in myocardial tissue for more than 10 days (Vong et al., 2018), and R gel was evaluated through 8 weeks while degrading more rapidly in an oxidative microenvironment (Wang et al., 2022). Local persistence can therefore be advantageous, but it also raises questions about residual material, degradation products, local inflammatory responses, mechanical integration, and eventual elimination.

Demonstration of payload release does not necessarily establish complete carrier degradation. A responsive bond can cleave while an inorganic component persists, or a matrix can release nanoparticles that remain in tissue. Terms such as ROS-degradable should therefore not be interpreted automatically as completely biodegradable and cleared unless degradation products and elimination are measured.

#### Platform-specific risk

The major material classes introduce different translational risks. Acellular polymeric or lipid nanoparticles require quantitative pharmacokinetic, biodistribution, clearance, and repeated-dose data. Local degradable hydrogels can reduce systemic exposure and improve retention but require assessment of invasive administration, myocardial persistence, degradation products, and local compatibility. Metal- and inorganic-containing nanozymes require additional evaluation of particle transformation, ion release, chronic deposition, and clearance (Fu et al., 2026; Gong et al., 2025; Wang et al., 2025c; Xu et al., 2026; Zhu et al., 2025).

Membrane-coated, exosome-derived, or biologically sourced carriers can improve inflammatory or myocardial targeting but add challenges involving source variability, composition, immunogenicity, storage, sterilization, and batch consistency (Li et al., 2026; Shiekh et al., 2022; Wang et al., 2025c; Wang et al., 2026; Zhu et al., 2024). Living-cell platforms introduce further concerns. The FAP-specific CAR T-cell hydrogel improved local cell infiltration and persistence while addressing poor myocardial trafficking and off-target toxicity associated with systemic administration [6]. Other systems delivered mesenchymal stem cells, hiPSC-derived cardiomyocytes, or brown adipose stem cells (Ding et al., 2022; Liu D. P. et al., 2026; Liu et al., 2023). Such therapies require long-term assessment of immune reactions, graft persistence, ectopic differentiation or tumorigenicity where relevant, and arrhythmogenicity.

Nucleic-acid platforms delivering miRNA, siRNA, or plasmid DNA require assessment of off-target gene regulation, innate immune activation, duration and reversibility of expression, and dose-dependent transcriptomic effects (Chen X. R. et al., 2025; Hu et al., 2026; Liao et al., 2025; Wang et al., 2025b). NO-, CO-, and H2S -releasing systems require specific control of systemic gas exposure, hemodynamic effects, repeated administration, dose-release relationships, and prolonged local exposure (Hao et al., 2022; Vong et al., 2018; Wang et al., 2026; Wang Y. et al., 2025; Wu C. R. et al., 2025). There is therefore no single generic nanomaterial safety question; safety requirements must follow the biological and physicochemical properties of each platform.

#### Large-animal validation remains a major bottleneck

Several studies extended cardiac assessment to 28 days or longer (Ikeda et al., 2016; Maranhao et al., 2017; Sun et al., 2023; Sun et al., 2024; Wang et al., 2025c; Wang et al., 2022; Zheng et al., 2024), which is important because acute reductions in ROS or infarct size do not establish durable effects on scar formation, ventricular remodeling, electrical stability, or material persistence. Large-animal validation remains rare. Study (Wang et al., 2025b) was the only explicitly identified porcine MI investigation, with 50-day follow-up and improvement in infarct size, cardiac function, muscle mass, and survival to scheduled harvest. This represents stronger translational evidence than rodent efficacy alone but does not replace chronic toxicology, rhythm monitoring, pharmacokinetic characterization, dose-ranging, or independent large-animal replication.

#### Manufacturing complexity increases with functional complexity

Several studies reported practical engineering features that may facilitate development, including rapid gelation, cell-free composition, relatively simple local-delivery formats, carriers described as stable or low-cost, and synthesis approaches proposed to facilitate scalability (Ding et al., 2020; Huang et al., 2022; Luo Q. H. et al., 2025; Tan et al., 2024). These are early engineering signals rather than manufacturing validation. Good Manufacturing Practice-compatible scale-up, validated sterilization, shelf life, storage stability, batch-release specifications, identity and potency assays, manufacturing yield, cost-of-goods analysis, and regulatory product classification were not identified systematically.

The burden increases as functional layers accumulate. A membrane-coated, mitochondria-targeted nanozyme carrying a therapeutic payload requires more identity, purity, loading, activity, stability, and reproducibility controls than a simpler acellular carrier. Greater multifunctionality should therefore not automatically be equated with greater translational value. A less complex platform that provides sufficient targeting and benefit with predictable manufacturing, administration, degradation, and safety may ultimately have a more plausible development pathway.

#### Overall translational position

Current evidence supports proof of concept for precision redox-directed cardiovascular delivery but not clinical readiness. The strongest translational signals include myocardial or cellular targeting, imaging-supported localization, selected pharmacokinetic improvements, responsive local retention, matched formulation controls, longer-term rodent efficacy, and an isolated large-animal validation (Hu et al., 2024; Ji et al., 2023; Li et al., 2026; Ma X. B. et al., 2025; Vong et al., 2018; Wang et al., 2025b; Wang et al., 2025c; Wang et al., 2022; Zhang et al., 2016). The principal weaknesses are incomplete whole-body biodistribution and clearance, sparse chronic cardiovascular safety testing, limited large-animal validation, heterogeneous degradation characterization, minimal repeated-dose evidence, modality-specific safety gaps, and little regulatory-grade manufacturing information.

Clinical applicability should therefore be judged not by the sophistication of ROS-responsive chemistry alone but by whether a platform provides an acceptable balance of therapeutic specificity, administration feasibility, material persistence, systemic exposure, cardiovascular safety, manufacturing reproducibility, and regulatory complexity. ROS-responsive cardiovascular biomaterials are translationally promising but clinically unproven. Platform-specific safety and translational considerations are summarized in Table 5, while Figure 5 illustrates the progressive translational-readiness gap from preclinical design and efficacy to human validation.

**TABLE 5:** Platform-specific safety and translational considerations.

Platform/risk domain	Current evidence signal	Main unresolved limitation	Translational implication	Refs
General systemic safety	Selected studies report no obvious toxicity, preserved organ function, or lower toxicity than systemic/free drug	Non-standardized toxicology; limited repeated-dose and chronic assessment	Supports preliminary tolerability only	(Fu et al., 2026; Ji et al., 2023; Lee et al., 2015)
Systemic targeted nanoparticles	Myocardial enrichment, reduced off-target sequestration, imaging and PK gains	Whole-organ biodistribution, clearance, dose proportionality, repeat dosing	Potential acute/peri-reperfusion applicability if PK and safety mature	(Li et al., 2026; Ma et al., 2025b; Wang et al., 2025c; Zhang et al., 2016)
Local hydrogels/patches	High myocardial retention, prolonged release, responsive degradation	Invasive administration, residual material, local toxicity, degradation products	Strong local-control potential but procedure-dependent applicability	(Ding et al., 2020; Tan et al., 2024; Vong et al., 2018; Wang et al., 2022)
Metal/inorganic nanozymes	Sustained catalytic activity and cardiac efficacy	Persistence, ion release, transformation, chronic deposition	Catalytic durability is both an advantage and a safety burden	(Fu et al., 2026; Gong et al., 2025; Wang et al., 2025c; Xu et al., 2026; Zhu et al., 2025)
Cells/biological carriers	Improved cell or vesicle retention and localized biological activity	Immunogenicity, graft persistence, arrhythmogenicity, source/batch variability	Higher regulatory and manufacturing complexity	(Cao et al., 2026; Ding et al., 2022; Liu et al., 2026a; Liu et al., 2023; Shiekh et al., 2022)
Nucleic-acid systems	miRNA, siRNA, or plasmid delivery broadens mechanism	Off-target regulation, innate immunity, duration and reversibility	Requires gene-therapy-specific safety characterization	(Chen et al., 2025b; Hu et al., 2026; Liao et al., 2025; Wang et al., 2025b)
NO/CO/H_2_S platforms	Local or targeted redox-gas delivery with therapeutic benefit	Dose-release control, systemic exposure, hemodynamic effects	High potential but narrow safety-control requirements	(Hao et al., 2022; Vong et al., 2018; Wang et al., 2026; Wang et al., 2025e; Wu et al., 2025a)
Large-animal/clinical maturity	One explicitly identified porcine MI study with 50-day follow-up	Sparse replication; no human evidence identified	Advanced preclinical signal, not clinical readiness	(Wang et al., 2025b)
Manufacturing readiness	Selected systems show rapid gelation, simple formulation, reproducibility, or proposed scalability	GMP scale-up, sterilization, stability, QC, potency, cost, and classification largely unreported	Manufacturing may limit highly multifunctional systems	(Ding et al., 2020; Huang et al., 2022; Luo et al., 2025b; Tan et al., 2024)

**FIGURE 5:**
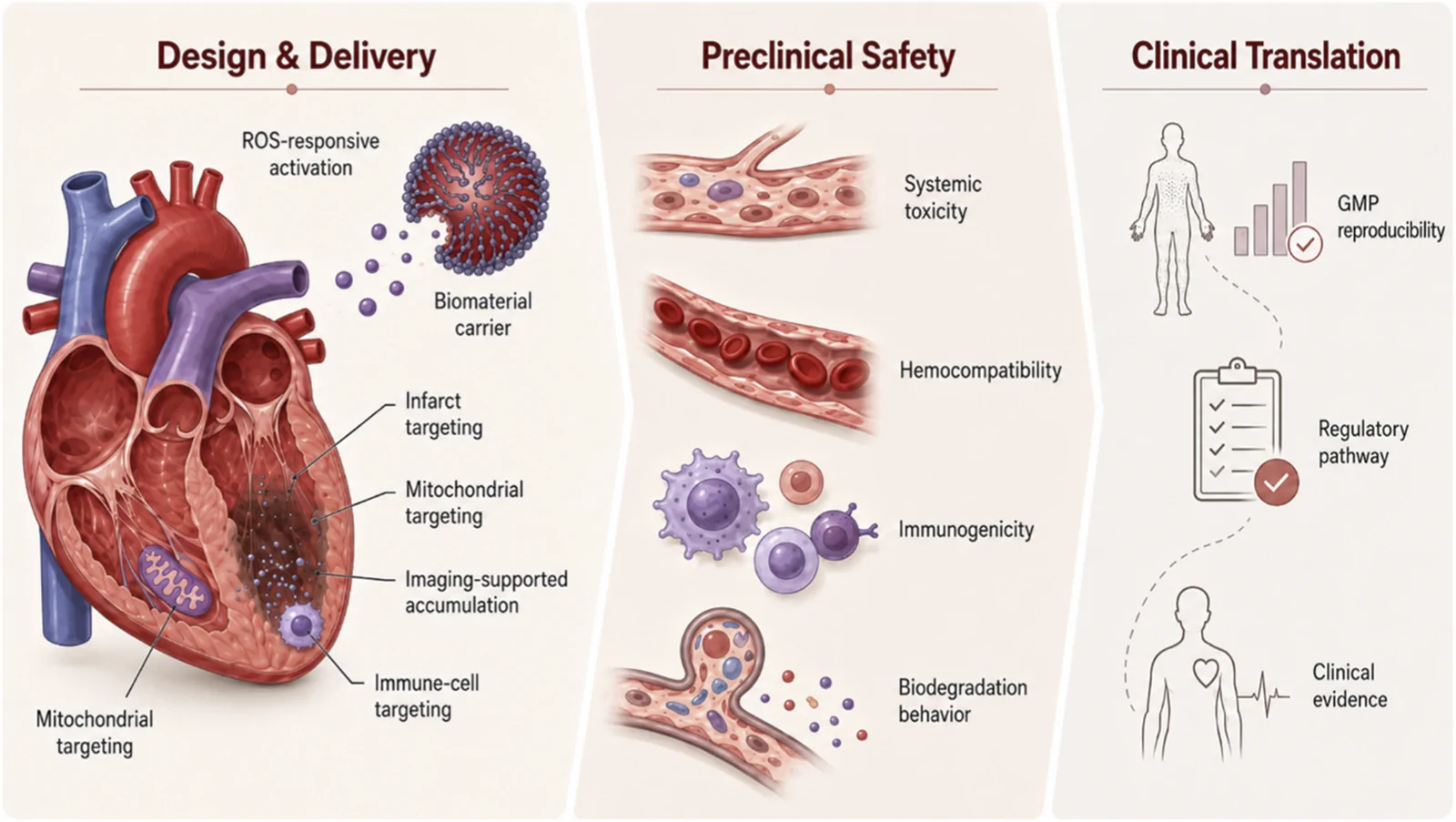
Translational-readiness gap for ROS-responsive cardiovascular biomaterials. The figure depicts a narrowing evidence pathway from strong preclinical material design and therapeutic efficacy toward progressively weaker evidence for quantitative pharmacokinetics and whole-body biodistribution, chronic cardiovascular safety, large-animal reproducibility, manufacturing control, and human validation. Platform-specific branches distinguish local hydrogels and patches, systemic nanoparticles and liposomes, metal or inorganic nanozymes, biologically derived carriers, cell and nucleic-acid therapies, and gas-delivery systems. Preliminary tolerability is separated from comprehensive safety evidence (*Prepared with the assistance of Gemini NotebookLM pro and OpenAI ChatGPT Plus*).

#### Limitations and future perspectives

The central limitation of the current evidence base is no longer a shortage of innovative ROS-interacting biomaterials, but a shortage of standardized evidence capable of distinguishing promising designs from clinically credible candidates. The field has generated diverse hydrogels, nanoparticles, liposomes, nanozymes, mitochondrial carriers, gas-delivery systems, and biological redox modulators, yet direct comparisons among these strategies remain rare. Future progress should prioritize depth of validation over continued expansion of platform variety.

#### Standardize what constitutes ROS-Responsive

The term ROS-responsive currently encompasses mechanistically distinct systems, including ROS-cleavable carriers, oxidatively degradable matrices, direct antioxidants, catalytic nanozymes, oxygen-generating biomaterials, redox-gas systems, and therapies that alter endogenous redox signaling (Cao et al., 2026; Ding et al., 2020; Hao et al., 2022; Song L. et al., 2025; Wang Y. et al., 2024; Xu et al., 2026; Zhen et al., 2025). Future studies should distinguish at minimum between ROS-triggered delivery, ROS-responsive degradation, direct ROS scavenging, catalytic ROS detoxification, and biological redox regulation. For truly stimulus-responsive systems, characterization should include activating species, physiologically relevant trigger concentrations, activation thresholds, release or degradation kinetics, payload stability, and matched low-ROS or non-responsive controls.

#### Replace indirect cross-study ranking with standardized comparative benchmarks

Current evidence does not support a definitive ranking of hydrogels, nanoparticles, nanozymes, or other platform classes because studies differ in species, ischemia duration, reperfusion protocol, MI induction, route, treatment timing, comparator groups, and follow-up (Ai et al., 2021; Huang et al., 2022; Ikeda et al., 2016; Mahmood et al., 2020; Maranhao et al., 2017; Shi et al., 2024). Future comparative studies should hold the therapeutic payload and disease model constant while varying one feature at a time, such as responsive versus non-responsive chemistry, targeted versus untargeted delivery, local versus systemic administration, or catalytic versus stoichiometric antioxidant activity. Existing studies of R gel versus non-reactive gel (Wang et al., 2022), NO-RIG versus simpler control gels (Vong et al., 2018), and MitoQ hydrogel by two administration routes (Tan et al., 2024) illustrate the value of this approach.

#### Match platform design to disease stage

MIRI and post-infarction remodeling should not be treated as a single therapeutic environment. Acute reperfusion is characterized by rapidly evolving oxidative and mitochondrial injury, whereas later repair involves inflammatory resolution, angiogenesis, fibroblast activation, scar maturation, and ventricular remodeling. Systemically deliverable nanoparticles or mitochondrial-targeted systems may be more compatible with narrow peri-reperfusion windows, whereas locally retained hydrogels, patches, fibroblast-directed systems, and staged-release platforms may be better aligned with post-MI repair. Apelin-13@Gel TK demonstrated different release behavior between high-ROS injury cores and lower-ROS border zones (Zhen et al., 2025), while SaB/Cand microgels linked early inflammatory control with longer-term antifibrotic therapy (Song L. et al., 2025). Future systems should therefore be designed around measured spatiotemporal redox biology rather than a generic assumption of uniformly high myocardial ROS.

#### Establish a stronger mechanistic hierarchy

Future studies should distinguish primary therapeutic mechanisms from downstream biological responses. A useful hierarchy is material or redox interaction, followed by proximal cellular mechanism, intermediate tissue response, and long-term cardiac outcome. ROS-responsive release, catalytic scavenging, or targeted mitochondrial delivery represents the primary intervention; mitochondrial stabilization, mPTP inhibition, calcium regulation, and suppression of regulated cell death are proximal effects; macrophage or inflammatory changes may be downstream or independently targeted; and fibrosis, angiogenesis, scar structure, ventricular remodeling, and function are later outcomes. The CsA nanoparticle study (Ikeda et al., 2016) demonstrates why this distinction matters: remodeling improved without altered inflammatory monocyte recruitment.

#### Make pharmacokinetics, biodistribution, and clearance core endpoints

Targeting sophistication has outpaced complete pharmacological characterization. Myocardial accumulation, cell selectivity, mitochondrial localization, and imaging-supported targeting have been demonstrated in selected systems (Hu et al., 2024; Li et al., 2026; Ma X. B. et al., 2025; Wang et al., 2025c; Zhang et al., 2016), yet circulation half-life, whole-organ biodistribution, dose proportionality, carrier-versus-payload disposition, degradation products, and excretion pathways remain inconsistently characterized. Future studies should integrate pharmacokinetic and pharmacodynamic analysis, including myocardial exposure, major off-target organ exposure, blood kinetics, target-to-nontarget ratios, and both carrier and payload clearance.

#### Elevate cardiovascular-specific safety to the same priority as efficacy

Most studies are designed to demonstrate cardioprotection rather than detect treatment-associated cardiovascular harm. Rhythm safety is particularly important for infarct-zone implants, conductive matrices, cell-based therapies, mitochondrial interventions, and prolonged local depots. Selected studies provide encouraging electrophysiological observations (Boarescu et al., 2019; Liu et al., 2016; Taiwaikuli et al., 2026), but systematic QT/QTc assessment, continuous rhythm monitoring, ventricular arrhythmia susceptibility, electrophysiological conduction, and delayed arrhythmogenicity remain limited. Platform-specific toxicology should be built into efficacy programs rather than deferred until after optimization.

#### Advance fewer candidates into rigorous large-animal validation

The evidence base is heavily dominated by rodents, whereas only study (Wang et al., 2025b) explicitly includes a porcine MI model. Future large-animal experiments should address clinically relevant myocardial scale, coronary anatomy, infarct heterogeneity, delivery feasibility, dose scaling, rhythm safety, systemic exposure, degradation, clearance, and chronic remodeling rather than simply reproducing rodent efficacy. Advancing a smaller number of well-characterized candidates through this pathway would provide greater translational value than continued proliferation of rodent-only platforms.

#### Prioritize manufacturable simplicity

Increasing multifunctionality creates a potential conflict between biological sophistication and product feasibility. Combining ROS-responsive bonds, multiple drugs, targeting peptides, biomimetic membranes, mitochondrial ligands, catalytic metals, imaging agents, and biological cargo can improve functionality but also increases manufacturing and regulatory burden. Future designs should determine whether each component provides measurable incremental benefit and remove components that do not improve localization, efficacy, safety, or dosing. Scalable synthesis, sterilization, storage stability, shelf life, batch reproducibility, loading consistency, potency assays, quality-control specifications, and regulatory classification should be considered early rather than after biological optimization.

#### Develop a precision-medicine framework for redox heterogeneity

A longer-term challenge is determining which patients, myocardial regions, and treatment windows provide sufficient redox conditions to activate a responsive platform. Clinical infarcts vary in duration, reperfusion success, infarct size, collateral circulation, comorbidities, medication exposure, and timing of presentation. The circadian HA-RES-OPC-MMP study showed that treatment efficacy varied when underlying injury severity differed between ZT1 and ZT13 (Zhang et al., 2024), while Apelin-13@Gel TK demonstrated different release behavior across ROS gradients within the same injured myocardium (Zhen et al., 2025). Future precision-delivery strategies may therefore require biomarkers or imaging approaches capable of identifying clinically relevant myocardial redox phenotypes.

#### Research priorities

Six priorities should define the next phase of the field: standardized definitions of ROS responsiveness; head-to-head comparator studies; disease-stage-matched experimental models; integrated pharmacokinetic, biodistribution, and mechanistic validation; platform-specific long-term cardiovascular safety; and scalable large-animal and manufacturing development. Progress across these areas would shift the field from demonstrating that sophisticated biomaterials can improve experimental myocardial injury toward determining which platforms provide sufficient incremental benefit under clinically realistic conditions to justify their added complexity and risk. The goal should not be maximal material complexity, but reproducible, disease-stage-specific redox intervention with a clear mechanism, measurable delivery advantage, durable cardiac benefit, predictable clearance, acceptable safety, and a credible manufacturing pathway.

## Concluding remarks

ROS-responsive and redox-interacting biomaterials provide a compelling preclinical strategy for converting the oxidative microenvironment of MIRI and post-infarction remodeling into a target for spatially and temporally controlled therapy. The evidence supports several distinct approaches, including ROS-triggered release or degradation, direct ROS scavenging, catalytic nanozyme activity, mitochondrial targeting, oxygen or redox-gas modulation, and biological redox-pathway regulation. These approaches should not be considered mechanistically or translationally equivalent.

The most informative platforms establish a coherent evidence chain from material-ROS interaction or targeting, through proximal mitochondrial or cell-death regulation, to inflammatory or reparative responses and finally to cardiac structural and functional outcomes. Importantly, this hierarchy is platform dependent. Mitochondrial protection can improve cardiac outcome without obligatory suppression of inflammatory recruitment (Ikeda et al., 2016), whereas other systems directly target macrophage, fibroblast, or immunometabolic pathways. Reductions in fibrosis, angiogenesis, or ventricular remodeling should therefore be interpreted as later tissue-level consequences unless direct mechanistic causality is demonstrated.

No single biomaterial class can presently be designated superior. Local hydrogels and microgels offer strong myocardial retention and opportunities for staged or ROS-gradient-responsive release (Song L. et al., 2025; Wang et al., 2022; Zhen et al., 2025) but often require invasive cardiac administration. Systemic nanoparticles and liposomes may be better suited to peri-reperfusion delivery and tissue-, cell-, or organelle-level targeting but require more complete pharmacokinetic, biodistribution, and clearance characterization. Catalytic nanozymes may provide sustained redox activity but introduce material-persistence and long-term safety questions. Cell-, nucleic-acid-, membrane-derived, and gas-delivery systems further expand therapeutic functionality while increasing manufacturing, safety, and regulatory complexity.

The quantitative evidence is strongest when interpreted within individual studies rather than as a cross-study ranking. Matched non-responsive carriers, free-drug controls, untargeted formulations, quantitative ROS-dependent release, pharmacokinetic analysis, and longitudinal cardiac endpoints provide stronger evidence than therapeutic improvement against saline alone. Direct head-to-head comparisons among major ROS-responsive platform classes remain largely absent.

Translation is considerably less mature than material design. The evidence base remains dominated by rodent models, with only one explicitly identified porcine MI study (Wang et al., 2025b) and no human observational or clinical-trial evidence identified. Long-term cardiovascular safety, arrhythmogenicity, complete biodistribution and clearance, degradation-product toxicity, repeat-dose effects, large-animal reproducibility, and regulatory-grade manufacturing remain incompletely characterized.

The next phase of the field should prioritize standardized definitions of ROS responsiveness, disease-stage-matched models, head-to-head comparator studies, quantitative pharmacokinetic and biodistribution analysis, platform-specific cardiovascular safety, rigorous large-animal validation, and manufacturable design. The most clinically credible system may ultimately be not the most multifunctional biomaterial, but the least complex platform that produces a reproducible delivery advantage, a clearly attributable mechanism, durable cardiac benefit, predictable clearance, and acceptable safety within a clinically realistic treatment workflow.

ROS-responsive cardiovascular biomaterials should therefore be regarded as mechanistically promising but clinically unproven precision-delivery technologies. Their future value will depend on whether sophisticated redox-responsive design can be translated into standardized, safe, scalable, and demonstrably superior therapy for acute reperfusion injury and post-infarction repair.

## Glossary

AUC: Area under the curve
CAR T: Chimeric antigen receptor T cell
**CeO**_**2**_: Cerium oxide
CHP: Cardiac homing peptide
CO: Carbon monoxide
COF: Covalent organic framework
CsA: Cyclosporine A
Cx43: Connexin 43
ECG: Electrocardiogram/electrocardiographic
EGCG: Epigallocatechin-3-gallate
FAP: Fibroblast activation protein
FDX1: Ferredoxin 1
GelMA: Gelatin methacryloyl
GLP: Good Laboratory Practice
GMP: Good Manufacturing Practice
GPX4: Glutathione peroxidase four
GSH: Glutathione
**H**_**2**_ **O**_**2**_: Hydrogen peroxide
**H**_**2**_ **S**: Hydrogen sulfide
hiPSC-CM: Human induced pluripotent stem cell-derived cardiomyocyte
LAD: Left anterior descending coronary artery
LDH: Layered double hydroxide
LVEF: Left ventricular ejection fraction
M1: Classically activated macrophage phenotype
M2: Alternatively activated/reparative macrophage phenotype
MI: Myocardial infarction
MIRI: Myocardial ischemia–reperfusion injury
miRNA: MicroRNA
MMP: Matrix metalloproteinase
MOF: Metal–organic framework
mPDA: Mesoporous polydopamine
mPTP: Mitochondrial permeability transition pore
MSC: Mesenchymal stem cell
mtDNA: Mitochondrial DNA
NF-κB: Nuclear factor kappa B
NO: Nitric oxide
Nrf2: Nuclear factor erythroid 2-related factor 2
**O**_**2**_: Oxygen
PBS: Phosphate-buffered saline
PCI: Percutaneous coronary intervention
PET/CT: Positron emission tomography/computed tomography
PLGA: Poly(lactic-co-glycolic acid)
QTc: Corrected QT interval
ROS: Reactive oxygen species
siRNA: Small interfering RNA
SOD: Superoxide dismutase
STEMI: ST-elevation myocardial infarction
TPP: Triphenylphosphonium
Treg: Regulatory T cellT cell
VEGF: Vascular endothelial growth factor
ZT: Zeitgeber time
